# Excess nitrogen responsive HvMADS27 transcription factor controls barley root architecture by regulating abscisic acid level

**DOI:** 10.3389/fpls.2022.950796

**Published:** 2022-09-12

**Authors:** Aleksandra Smoczynska, Andrzej Pacak, Aleksandra Grabowska, Dawid Bielewicz, Marcin Zadworny, Kashmir Singh, Jakub Dolata, Mateusz Bajczyk, Przemyslaw Nuc, Jacek Kesy, Magdalena Wozniak, Izabela Ratajczak, Wendy Harwood, Wojciech M. Karlowski, Artur Jarmolowski, Zofia Szweykowska-Kulinska

**Affiliations:** ^1^Department of Gene Expression, Institute of Molecular Biology and Biotechnology, Faculty of Biology, Adam Mickiewicz University, Poznań, Poland; ^2^Center for Advanced Technology, Adam Mickiewicz University, Poznań, Poland; ^3^Institute of Dendrology, Polish Academy of Sciences, Kórnik, Poland; ^4^Department of Biotechnology, Panjab University, Chandigarh, India; ^5^Institute of Biology, Faculty of Biological and Veterinary Sciences, Nicolaus Copernicus University, Toruń, Poland; ^6^Department of Chemistry, Faculty of Forestry and Wood Technology, Poznan University of Life Sciences, Poznań, Poland; ^7^Department of Crop Genetics, John Innes Centre, Norwich Research Park, Norfolk, United Kingdom; ^8^Department of Computational Biology, Institute of Molecular Biology and Biotechnology, Adam Mickiewicz University, Poznań, Poland

**Keywords:** miRNA, nitrogen, barley, root architecture, abscisic acid, HvMADS27

## Abstract

Nitrogen (N) is an important element for plant growth and development. Although several studies have examined plants’ response to N deficiency, studies on plants’ response to excess N, which is common in fertilizer-based agrosystems, are limited. Therefore, the aim of this study was to examine the response of barley to excess N conditions, specifically the root response. Additionally, genomic mechanism of excess N response in barley was elucidated using transcriptomic technologies. The results of the study showed that barley MADS27 transcription factor was mainly expressed in the roots and its gene contained N-responsive *cis*-regulatory elements in the promoter region. Additionally, there was a significant decrease in *HvMADS27* expression under excess N condition; however, its expression was not significantly affected under low N condition. Phenotypic analysis of the root system of *HvMADS27* knockdown and overexpressing barley plants revealed that HvMADS27 regulates barley root architecture under excess N stress. Further analysis of wild-type (WT) and transgenic barley plants (*hvmads27 kd* and *hvmads27 c-Myc OE*) revealed that HvMADS27 regulates the expression of HvBG1 β-glucosidase, which in turn regulates abscisic acid (ABA) level in roots. Overall, the findings of this study showed that *HvMADS27* expression is downregulated in barley roots under excess N stress, which induces *HvBG1* expression, leading to the release of ABA from ABA-glucose conjugate, and consequent shortening of the roots.

## Introduction

Nitrogen (N) availability is a primary factor affecting the size and quality of crop yield (Anas et al., 2020). Over the years, intensive crop production has led to increased fertilizer use, resulting environmental problems (Goulding et al., 2008; Jiao et al., 2016; Al-Tawaha et al., 2021). Studies have extensively examined plant response to N deficiency (Vos et al., 2005; Uribelarrea et al., 2009; Kant et al., 2011; Chen et al., 2015; Mu et al., 2016; Xiong et al., 2018); however, studies on plants response to high N supply are limited. Under excess N conditions, it is known that plants exhibit severe phenotypes, including reduced inflorescence, short roots, yellowing of leaves, reduced grain yield, and high susceptibility to pathogens (Lehmeier et al., 2013; Kong et al., 2017; Grabowska et al., 2020). Thus, illuminating the mechanisms of excess N response in barley (*Hordeum vulgare*), an economically important crop ranking fourth in global production, could improve fertilizer use efficiency in agroecosystems (Newton et al., 2011; Chaudhry et al., 2021).

Nitrate (NO_3_^−^) and ammonium (NH_4_^+^) ions are absorbed by the root system using specific transporters. Two nitrate uptake systems formed by LOW and HIGH-AFFINITY TRANSPORTERS (LATS and HATS, respectively) were identified. LATS is encoded by the *NITRATE TRANSPORTER 1/PEPTIDE TRANSPORTER* (*NRT1/PTR*) family genes (*NPF*), which includes 53 members in *Arabidopsis,* 93 in rice, and only three members have been identified in barley based on their similarity to rice proteins (*HvNRT1.1*, *HvNRT1.2*, and *HvNRT1.5*; Leran et al., 2014; Wittgenstein et al., 2014; Liu et al., 2020). HATS are encoded by the *NITRATE TRANSPORTER 2* (*NRT2*) gene family, and only seven members have been identified in Arabidopsis, five in rice, and four in barley (Chopin et al., 2007; Feng et al., 2011). NITRATE TRANSPORTER 1 (NRT1.1), which acts as a N sensor, is the most studied member of the LATS, and is exclusively expressed in roots (Ho et al., 2009). *NITRATE TRANSPORTER 2* (*NRT2*) genes encode HATS that act under low soil N conditions, and interacts with NITRATE ASSIMILATION RELATED PROTEIN (NAR2) family members, for proper functioning (Orsel et al., 2006). AMMONIUM TRANSPORTERS (AMTS) are involved in the absorption of ammonium ions (Ho et al., 2009). Presently, six AMTS genes have been identified in Arabidopsis, 10 in rice, and 16 in soybean (Gazzarrini et al., 1999; Sonoda et al., 2003; Kobae et al., 2010). Nitrate and ammonium ions absorbed by plants are further incorporated into amino acids. Ammonium is the most preferable source of nitrogen for plants, because nitrate needs to be reduced to ammonium, which requires more energy (Bloom et al., 1992; Filiz and Akbudak, 2020). First, nitrate is reduced to nitrite in the cytosol by nitrate reductase (Meyer and Stitt, 2001), and then translocated to the chloroplasts where it is reduced to ammonium by nitrite reductase (Meyer and Stitt, 2001). Ammonium is then assimilated in the GS/GOGAT cycle, where glutamine synthetase (GS) fixes ammonium on glutamate to form glutamine. Subsequently, glutamine 2-oxoglutarate amino transferase (GOGAT) catalyzes the reaction of glutamine with 2-oxoglutarate to form two molecules of glutamate (Lea and Miflin, 1974; Lea and Forde, 1994; Unno et al., 2006). Other amino acids are the result of transamination reactions in which glutamine is the substrate (Tcherkes and Hodges, 2008).

Several studies have examined the relationship between nitrogen metabolism in plants and the production of phytohormones, particularly abscisic acid (ABA; Finkelstein et al., 2002; Cutler et al., 2010; Yan et al., 2014; Sah et al., 2016; Harris and Ondzighi-Assoume, 2017; Jiao et al., 2020). ABA regulates several processes in plants, including seed germination, abiotic and biotic stress responses, and primary root growth (Finkelstein et al., 2002; Cutler et al., 2010; Yan et al., 2014; Sah et al., 2016; Jiao et al., 2020). In Arabidopsis, an increase in environmental N in the rhizosphere causes gradual accumulation of ABA in roots, and N induces ABA-responsive genes (Harris and Ondzighi-Assoume, 2017). In Arabidopsis, SCARECROW (SCR) transcription factor (TF) controls root elongation by repressing the expression of *ABA INSENSITIVE 4* (*ABI4*) and *ABA INSENSITIVE 5* (*ABI5*) transcription factor genes. Interestingly, it has been shown that *SCR* expression is controlled by both N and ABA (Harris and Ondzighi-Assoume, 2017). Although root development and growth are potentially beneficial for N uptake from soil (Wiren et al., 1997), an in-depth understanding of different traits and their drivers is essential to determine the process affecting N acquisition (Frescher et al., 2020; Rehman et al., 2021).

Recently, Wang et al. (2020) reported that the TaANR1-MADS-box transcription factor regulates ABA level in wheat roots by activating *TaBG1* β-glucosidase and regulating the expression level of *TaWabi5* transcription factor gene that controls the expression of wheat nitrogen transporter genes from the *TaNRT2* family. MADS-box transcription factors have been shown to play an important role in plant root responses to N and are conserved in nearly all eukaryotes (Yan et al., 2014; Yu et al., 2015; Zhang et al., 2018; Wang et al., 2020). MADS-box name is derived from the yeast MINICHROMOSOME MAINTENANCE 1 (MCM1; Passmore et al., 1988), the Arabidopsis AGAMOUS (AG; Yanofsky et al., 1990), the Antirrhinum DEFICIENS (DEFA; Schwarz-Sommer et al., 1990), and the mammalian SERUM RESPONSE FACTOR (SRF) proteins (Norman et al., 1988).

MADS-box domain consists of 58 amino acid residues at the N-terminus that binds to a consensus CC [A/T]_6_GG sequence, termed as the “CArG-box” motif (Hayes et al., 1988; Riechmann et al., 1996; Guo et al., 2013). Structure of MADS-box transcription factors consists of four protein domains: DNA binding MADS-box (M) domain, the less-conserved intervening domain (I) that ensures interaction specificity between different MADS-box transcription factors and/or other proteins, the keratin-like coiled-coil (K) domain for conferring protein–protein interactions, and a highly variable C-terminal (C) domain for regulating gene transcription or protein complexes formations (Hill et al., 2008).

Yan et al., 2014 reported that rice *OsMADS23,25,27,57,* and *61* regulate lateral root elongation in response to N and are regulated by miRNA444a. Overexpression of this miRNA results in reduced lateral root elongation, but promotes primary and adventitious root growth in an N-dependent manner. Moreover, studies have shown that *OsMADS25*, which is specifically expressed in rice roots, regulates root development and affects plant N accumulation (Yu et al., 2015; Zhang et al., 2018). Although we have already knowledge on the role of rice MADS TFs and miRNAs from miR444 family in root response to N, nothing is known about the mechanisms of root response to N in barley plants.

Thus, the aim of this study was to reveal the role of selected MADS TF and miR444 family members in shaping barley root architecture in response to N. In this paper, we reveal a novel molecular mechanism of barley root shortening in response to N excess stress. We present the findings that *HvMADS27* expression is downregulated in barley roots under excess N stress, which induces *HvBG1* expression, leading to the release of ABA from ABA-glucose conjugate, and consequent shortening of the roots. It seems that miR444 family members do not exert the major role in the downregulation of *HvMADS27* expression under N excess stress.

The results of our studies may be relevant for plant scientist working on plant response to various environmental conditions and for breeding companies working on developing new strategies to engineer crop plants resistant to various environmental cues (Imran et al., 2021).

## Materials and methods

### Plant material and growth conditions

Seeds of spring barley (*H. vulgare* cultivar Golden Promise) were germinated on wet sterile Whatman filter paper for 3 days in a growth chamber (MLR35−1H, Sanyo, Panasonic) under a 16/8 h light/dark photoperiod at 20°C until white hypocotyls were visible. Two seedlings were then transferred to a single pot containing perlite supplemented with medium containing 28 mM NH_4_NO_3_, 20 mM KH_2_PO_4_, 4 mM K_2_SO_4_, 16 mM MgSO_4_:7H_2_O, 53 μM H_3_BO_3_, 8 μM CuSO_4_, 4 μM MnSO_4_:H_2_O, and 120 μM FeCl_3_:6H_2_O (Pacak et al., 2016a). To create high N stress condition, the concentration of NH_4_NO_3_ in the medium above was increased by 10 times, which falls within the range of concentrations recommended in agriculture (Lips et al., 1990; Yao et al., 2011; Heuermann et al., 2021). The expression of the *HvNRT1.1* transporter gene was used as a marker of N stress (Supplementary Figure S3). The plants were subsequently grown under controlled conditions (20°C day temperature and 15°C night temperature) under a 16/8 h light/dark photoperiod.

### RNA isolation and quantitative PCR

Total RNA was extracted from the roots using the Direct-zol RNA Mini Prep Kit (Zymo Research), according to the manufacturer’s instructions. RNA quality and quantity were assessed using NanoDrop ND-1000 spectrophotometer, and RNA integrity was estimated on a 1.2% agarose gels. Thereafter, 3 μg of DNA-free RNA was reverse-transcribed to generate cDNA using SuperScript III Reverse Transcriptase (Invitrogen, Carlsbad, CA, United States) and oligo(dT)_18_ (Novazym, Poland) primers. cDNA samples were diluted 4-times and 1 μl was used as template. Quantitative real-time PCR was performed using Power SYBR® Green PCR Master MIX (Applied Biosystems, Warrington, United Kingdom) and two primers specific for the gene of interest (for example AS104 and AS105, respectively, when expression of *HvMADS27* was analyzed; see Supplementary Table S2). Final concentration 500 nM each on a 7900HT Fast Real-Time PCR System (Applied Biosystems) in 10 μl reaction volumes in 384-well plate. The PCR conditions were as follows: 10 min at 95°C, 40 cycles for 15 s at 95°C, and 1 min at 60°C. Each real-time PCR reaction was performed independently for the three biological replicates. The barley *ARF 1* (*ADP-RIBOSYLATION FACTOR 1-like*; GenBank: AJ508228.2) gene fragment of 61 nt was simultaneously amplified and detected as an internal reference (Grabowska et al., 2020).

### RACE analysis

To analyze *HvMADS27* and *HvMIR444c* gene structure, 5′ RACE experiments were conducted using SMARTer® RACE cDNA Amplification Kit (Takara Bio, United States, Cat# 634860) and Advantage Polymerase as described by Kruszka et al. (2014). PCR products were cloned into the pGEM T-Easy vector (Promega) and sequenced (Faculty’s Laboratory of Molecular Biology Techniques, Adam Mickiewicz University in Poznan, Poland).

### Small RNA and transcriptomic library preparation

sRNA data of barley roots in the control and high N stress treatment groups used in this study were previously published by Grabowska et al. (2020) and deposited in the GO database under accession number GSE145799.

For transcriptomic analysis, total RNA was extracted from the root of WT and *HvMADS27* knockdown plants as described above and analyzed with RNA Nano Chips and Agilent Bioanalyzer System (Agilent Technologies, USA, No. 5067-1511). Samples with RIN numbers above 8 were further subjected to rRNA depletion using the RiboMinus Plant Kit for RNA-Seq (Invitrogen, United States, No. 2157461), and RNA concentration was further assessed using Qubit 3.0 Fluorometer (Invitrogen, United States). Approximately, 100 ng of RNA was used per library. Sequence libraries were prepared using NEB Ultra II Directional RNA Library Prep kit for Illumina (New England BioLabs, United States, No. E7760S), according to the manufacturer’s instructions and the quality of the prepared libraries was assessed using an Agilent Bioanalyzer DNA Chip (Agilent Technologies, No. 6067-1504, United States). Thereafter, samples were sequenced on Illumina platform. Sequence quality was assessed using the FASTQC program, and adaptor trimming was performed using Trimmomatic software. All obtained reads were mapped to the barley genome and transcriptome using HISAT2 and SALMON programs, respectively, and statistical analysis was performed in R studio environment using DESeq2 software (Love et al., 2014).

Obtained data are available under accession number: PRJNA746688.

### PARE library construction

Barley Parallel Analysis of RNA Ends (PARE) library was constructed using protocol described by German and colleagues (German et al., 2009). For barley PARE library construction instead of MmeI restriction enzyme, we used EcoP15I restriction enzyme for generation of 26–27 bp fragments as described previously (Kruszka et al., 2014; Alaba et al., 2015). RNA and DNA adapters were modified for compatibility to the TruSeq sequencing system of Illumina. Poly(A)-enriched RNA was isolated from barley line Rolap harvested at 68-day-old stage (kernel formation), described by as previously (Pacak et al., 2016b). PARE library was sequenced using Illumina technology in Fasteris SA (Switzerland) as it was described previously (Alaba et al., 2015). NGS sequences were trimmed and analyzed using CLC Genomics Workbench (QIAGEN Aarhus A/S). T-plots showing microRNA directed cleavage sites were constructed using PAREsnip2 software (Thody et al., 2018).

### Generation of transgenic lines

The WMD3-Web MicroRNA Designer^1^ was used to construct transgenic lines overexpressing artificial miRNA amiRMADS27 that targets *HvMADS27* mRNA. From the ranking list, we chose miRNAs with the best qualities and ordered the GeneArt gateway-compatible vector containing artificial miRNA/miRNA* embedded in barley pre-miRNA167h body where miRNA167h and its cognate miRNA* were replaced accordingly (Kruszka et al., 2013; Zielezinski et al., 2015; see Supplementary Figure S4). Finally, we performed LR cloning using LR clonase according to the manufacturer’s instructions, and cloned pre-miRNA167h with amiRMADS27 cassette into the designated vector, pBRACT214.^2^

To construct transgenic lines overexpressing *HvMADS27* with c-Myc tag, mRNA of *HvMADS27* was amplified on cDNA template from 2-week-old barley roots with primers containing restriction sites for EcoRI and BamHI, respectively. After restriction digestion at 37°C for 1 h, insert was cloned into the pGBKT7 plasmid containing the c-Myc tag. Then, we amplified *HvMADS27* cDNA with a c-Myc tag DNA insert with a forward primer containing CACC and a Kozak sequence at the 5′ end, reverse primer from the previous step, and cloned the insert into the pENTR plasmid. Finally, we performed cloning with LR clonase to the destination vector-pBRACT214.

To produce transgenic barley plants, we transformed *Agrobacterium tumefaciens* AGL1 strain with prepared constructs and performed barley immature embryo transformation at the John Innes Centre in Norwich according to the protocol of Hinchliffe and Harwood (2019).

Plants from a T0 generation were genotyped and the presence of a transgene was confirmed using PCR. In the next step, from four independent transgenic lines 100 plants of T1 generation were sown and further genotyped for the presence of the transgene as well as Northern blot experiments were performed for lines expressing artificial miRNA and western blot for lines expressing *HvMADS27* with c-Myc tag. Expression level of *HvMADS27* was analyzed and plants that exhibited the lowest and highest *HvMADS27* mRNA levels, respectively, were further bred. T2 generation plants were used for subsequent experiments.

### Root structure analysis

To analyze the root structure of WT plants, *hvmads27* knockdown lines, and *hvmads27 c-Myc* overexpressing lines plants were grown for 2 weeks and collected. Plants representing four transgenic lines were used for the measurements. From each transgenic line 20 plants were used. Roots were cut off, placed in a tray with water, and scanned using an Epson scanner at 300 dpi resolution and WinRHIZO software (Regent Instruments, Inc., Quebec, Canada). The total root length was further examined. Data obtained were normalized using Shapiro–Wilk test, and analyzed using Statistica software 13.1 (Kraków, Poland). Mean comparison was performed using Kruskal–Wallis test, *p < 0.05, **p < 0.01, and ***p < 0.0001.

### Measurement of N and ABA levels

Plants for the N and ABA determination were grown under the conditions described above. However, for treatment with ABA synthesis inhibitor, we supplemented the medium with 100 μM norflurazon (Sigma cat num: 34364; Dong et al., 2013; Wang et al., 2020). Approximately, 200 mg of pulverized root was incubated overnight in a cold room (11°C) in a mixture of 80% acetonitrile with 5% formic acid and 1 mM BHT and an internal standard, deuterated ABA (10 ng/sample). Then, magnesium sulfate and sodium chloride (1:3) were added, and samples were vortexed for 1 min and then centrifuged for 8 min at maximum speed (14,000 × *g*). Sodium sulfate was added to the obtained supernatants, and the samples were vortexed and centrifuged as described above. The obtained supernatant was dried under a stream of nitrogen at 45°C and the remaining residue was suspended in 1 M formic acid. The samples were then extracted using solid-phase C18 octadecyl columns (J.T. Baker C18 columns #7020–01), dried under a stream of nitrogen at 45°C, and the remaining residue was suspended in 100 μl of 80% methanol, followed by suspension in 35% methanol.

The ABA concentration of the roots was determined using ultra-pressure liquid chromatography tandem mass spectrometry (UHPLC–MS/MS; Shimadzu Nexera XR UHPLC/LCMS-8045 system; Kyoto, Japan) equipped with an Ascentis Express C-18 column (2.7 μm, 100 × 2.1 mm, Supelco, United States). Then, the obtained peak surfaces of standard and endogenous ABA were exported to an Excel file and analyzed using the formula: (endogenous ABA peak surface/internal standard surface) × 10/weight of used tissue in grams. The obtained results were divided by a coefficiency factor of 0.98 and subjected to statistical analysis using Student’s *t*-test *p < 0.05, **p < 0.01, and ***p < 0.0001.

The N content of the samples was determined using a Flash 2000 elemental analyzer (Thermo Fisher Scientific, USA). Instrument calibration was performed using standard BBOT [2,5-bis- (tert-butyl-benzoxazol-2-yl) thiophene; Thermo Fisher Scientific, United States] and Alfalfa certified reference material (Elemental Microanalysis Ltd., United Kingdom). Six-point calibration curves were plotted for each element (C, H, N, S) using the K factor as the calibration method (Rybak et al., 2020).

### ABA sensitivity assay

Germinated seedlings of WT plants, plants overexpressing *HvMADS27* with c-Myc tag and *HvMADS27* knockdown plants were grown on ½ MS plates (four seeds per plate, and three plates for variant) in a growth chamber (MLR35−1H, Sanyo, Panasonic) under long-day conditions at 20°C with supplementation with either 0.1 M NaOH in the control condition or 50 μM ABA dissolved in 0.1 M NaOH for ABA treatment. After a week, measurements of plant roots were taken and analyzed statistically using Excel software, and a standard *t*-test.

### Western blot analysis

Protein extracts for western blot analysis were prepared using buffer containing 50 mM Tris–HCl pH 7.5, 100 mM NaCl, 0.25% Triton X-100, 1 mM EDTA, 10 mM NaF, 1 mM Na_3_VO_4_, 0.25% NP-40, 1 mM PMSF, 1x protease inhibitor complete EDTA-free (Roche), and 10 μM MG132. Protein extracts were separated by 13% SDS-PAGE, transferred to a polyvinylidene difluoride membrane (PVDF; Millipore), and analyzed by western blotting using antibodies at dilutions recommended by the manufacturer: anti-c-Myc HRP (9E10 cat num# MA1-980-HRP) anti-BG1 (Agrisera, AS20 4419), anti-H3 (Abcam, ab18521), and anti-rabbit (Agrisera, AS09 602).

### Northern blot analysis

RNA electrophoresis, blotting, and hybridization were performed as previously described by Kruszka et al. (2013).

### ChIP-qPCR

ChIP was performed as previously described by Dolata et al. (2015) using 2 g per biological replicate of pulverized root sample from WT plants and plants overexpressing *HvMADS27 c-Myc*. Approximately 2 μg of anti-c-Myc (Agrisera, AS15 3035) antibody per IP was used. For control, root samples from mutants without the addition of antibody and WT plants with the addition of anti-c-Myc antibody was used. Thereafter, IP samples were washed, eluted, and incubated with proteinase K for 6 h at 65°C and used as a template for RT-qPCR, and primers flanking used are listed Supplementary material.

### Bioinformatic tools

Genomic sequences for *HvMADS27* and *HvMIR444c* were obtained from the Ensembl Plants database^3^ and aligned with the cDNA sequence using MAFFT version 7.^4^ Results from transcriptome sequencing were quality checked using the FASTQC program, and adaptor trimming was performed using Trimmomatic software. All obtained reads were mapped to the barley genome and transcriptome using HISAT2 and SALMON programs, respectively, and statistical analysis was performed in R studio environment using DESeq2 software (Love et al., 2014). Thereafter, the online tool WMD3-Web MicroRNA Designer^5^ was used to prepare construct overexpressing artificial miRNA (amiRMADS27) that targets *HvMADS27* mRNA. Root systems were analyzed using an Epson scanner and WinRHIZO software (Regent Instruments, Inc., Quebec, Canada). Total root length was further analyzed statistically using Statistica software 13.1 (Kraków, Poland). Densitometric analysis of western blots was performed using Image Studio Lite program version 5.2.5, and the amount of BG1 protein was normalized using WT control to normalize all bands (treated as 1.0, LI-COR, United States).

### Arabidopsis and barley protoplasts transfection and HvMADS27 localization

Berley leave protoplasts were isolated as described in (Frank et al., 2019) with minor modifications: Leaves of 1-week-old barley seedlings were cut and epiderma was removed. Leaves were incubated in enzyme solution containing cellulase “Onozuka R-10” (#16419.05, Serva, Germany) and macerozyme R-10 (#M8002.0010 Duchefa Biochemie, Netherlands) in darkness in 28°C for 80 min. After that time buffer containing protoplasts was filtered using nylon filter (#NY8004700, Millipor) and centrifuged. Next, the gradient was prepared with creating a sublayer of floating buffer pH 5.8 (1 mM CaCl_2_x2H_2_0, 40 mM sucrose, 100 nM sorbitol, and 5 mM MES) and upper layer of W5 buffer pH 5.8 (154 mM NaCl, 125 mM CaCl_2_x2H_2_0, 5 mM KCl, and 5 mM glucose). Further, the middle layer containing live protoplasts was collected and then suspended in Wash Solution (500 mM sorbitol, 20 nM KCl, and 4 mM MES). Transformation was performed using 100 μl of protoplasts and two plasmids: one containing *GFP-HvMADS27* and one *RFP-SERRATE* using 40 %PEG in 1x MaMg solution. Samples were incubated overnight and observed under the microscope. SERRATE was used as a nuclear marker (Yang et al., 2006).

The isolation of protoplasts from Arabidopsis was done exactly as described previously (Bajczyk et al., 2020). Localization of MADS27-GFP and RFP-SERRATE in both Arabidopsis and barley protoplasts was done using a Nikon A1RSi Inverted Confocal Microscope using 40×/1.25 water-immersion objectives. Excitation was achieved with an Ion argon laser at 488 nm (GFP) and with a diode laser at 561 nm (RFP). Fluorescence was observed using the emission spectrum range of 525/50 nm (GFP) and 595/50 (RFP).

## Results

### *HvMADS27* is expressed mainly in roots and is responsive to high N stress

**Figure 1:**
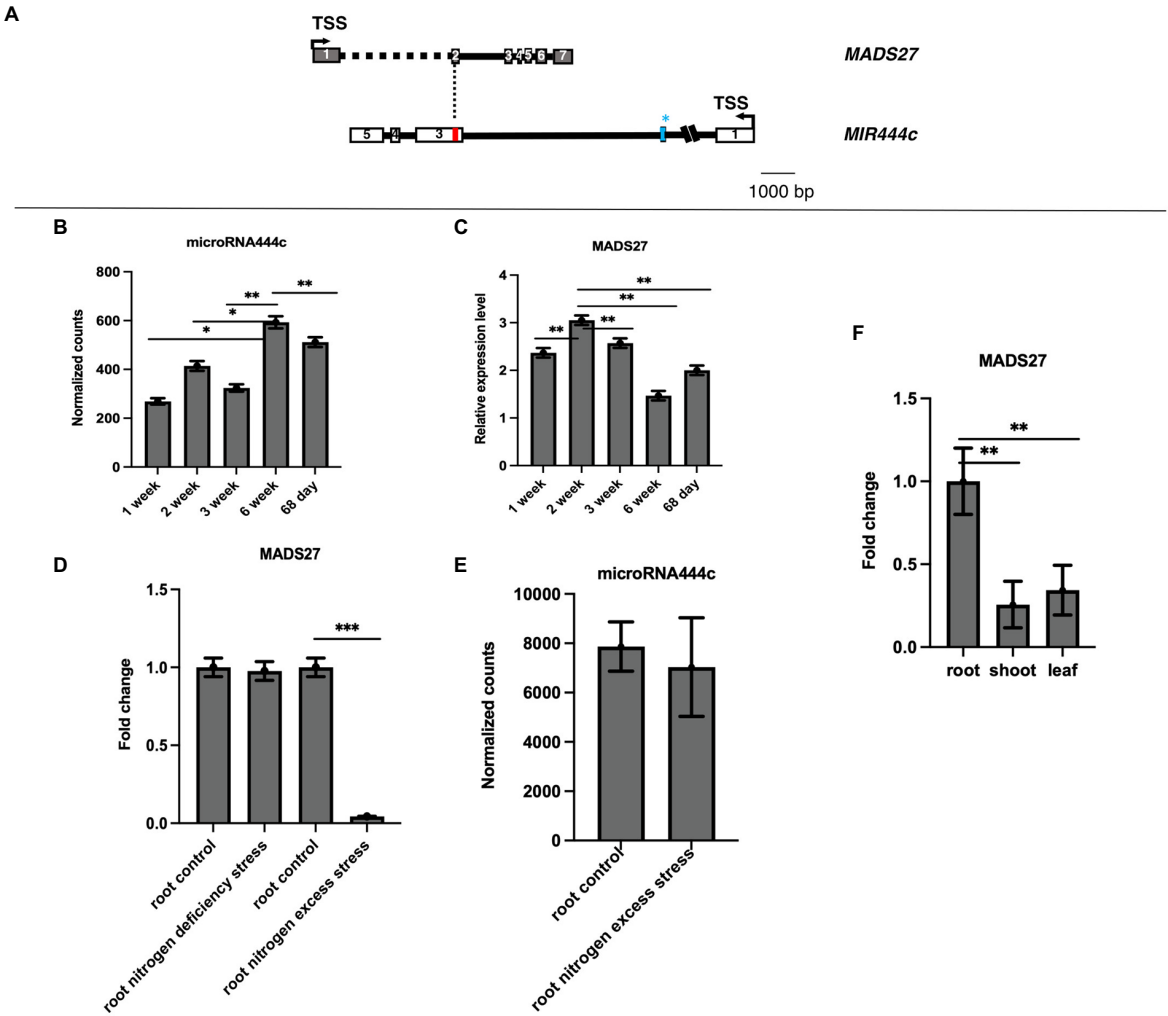
Gene structure and expression pattern of *HvMADS27* and *HvmiRNA444c*. **(A)** *HvMADS27* and *MIR444c* gene structures based on the 5′ and 3′ RACE analysis. White and gray blocks represent exons and black lines introns, blue bar in the secondexon of *MIR444c* gene represents miRNA444c^✶^ and red bar in the third exon represents miRNA444c, while dotted line marks overlap between two genes. **(B)** Results of deep sequencing of sRNA libraries, where the *x*-axis represents barley developmental stages and *y*-axis normalized counts. Mature miRNA444c occurred at the highest expression in sixth week of development. **(C)** *HvMADS27* mRNA relative to *ARF 1* mRNA level (Gene Bank: AJ508228.2) expression in barley at different developmental stages, with the *x*-axis representing developmental stages and y-axis expression of *HvMADS27*. The highest expression was observed at the second week of barley development. **(D)** RT-qPCR data showing *HvMADS27* expression in the roots of 2-week-old barley under N scarcity and excess N stress. There was a significant downregulation in *HvMADS27* expression under excess N stress, but no change was observed under N scarcity. **(E)** Level of mature miRNA444c based on deep sequencing of sRNA analysis in roots of plants grown under control and excess N conditions. No change was observed in the expression level of mature miRNA444c under excess N stress conditions compared with the control. *p* value based on Bonferroni correction was used. **(F)** RT-qPCR results showing that *HvMADS27* is mainly expressed in the roots of 2-week-old barley plants. **(D)** Student’s *t*-test was applied; *p < 0.05, **p < 0.01, ***p < 0.0001.

The gene structure and genome position of *HvMADS27* was analyzed to determine its function. The 5′ and 3′ RACE experiments revealed that the *HvMADS27* gene contains seven exons and six introns, and is located on the second chromosome (HORVU2Hr1G080490) on the DNA strand opposite to the *MIR444c* gene. After transcription and transcript maturation, there was approximately 60 nucleotides overlap between them, including perfect complementarity between miR444c and target *HvMADS27* mRNA (Figure 1A). Furthermore, degradome analysis of 68-day old samples of barley grown under control growth conditions showed that *HvMADS27* mRNA is likely targeted by miR444c (Supplementary Figure S1). Deep sequencing of small RNAs from selected barley developmental stages revealed that miRNA444c was highly expressed in the 6th week of development (Figure 1B). RT-qPCR analysis showed that the highest expression of *HvMADS27* was during the second week of barley growth (Figure 1C). Furthermore, *HvMADS27* was mainly expressed in the roots of two-week-old barley plants, necessitating further focus on this organ (Figure 1F; Supplementary Figure S2). Analysis of GFP-HvMADS27 localization in barley and Arabidopsis protoplasts revealed nuclear localization of the protein (Supplementary Figure S3). To elucidate the biological function of *HvMADS27* gene, regulatory motifs on the promoters of both *MIR444c* and *HvMADS27* were examined, and the results showed several common elements connected to the N response (Supplementary Table S1). Additionally, analysis of the expression of *HvMADS27* in the roots under N deprivation and excess conditions showed that there was a considerable downregulation in the expression of *HvMADS27* mRNA under excess N stress and not under N deprivation conditions (Figure 1D). However, the correlation of *HvMADS27* mRNA level in roots under N excess with expression of mature miRNA444c revealed no significant changes in the expression of mature miRNA444c, suggesting that this miRNA was not involved in *HvMADS27* regulation, at least under excess N stress (Figure 1E). Physiological analysis showed that plants grown under N deprivation conditions were characterized by yellowing leaves and longer roots in comparison with those grown under normal condition. In contrast, plants grown under excess N condition exhibited shorter roots compared with those of plants grown under normal condition (Supplementary Figure S4).

### *HvMADS27* controls the root architecture of barley: Mutants with knockdown of *HvMADS27* display shorter roots while mutants overexpressing *HvMADS27* show similar phenotype to WT plants

**Figure 2:**
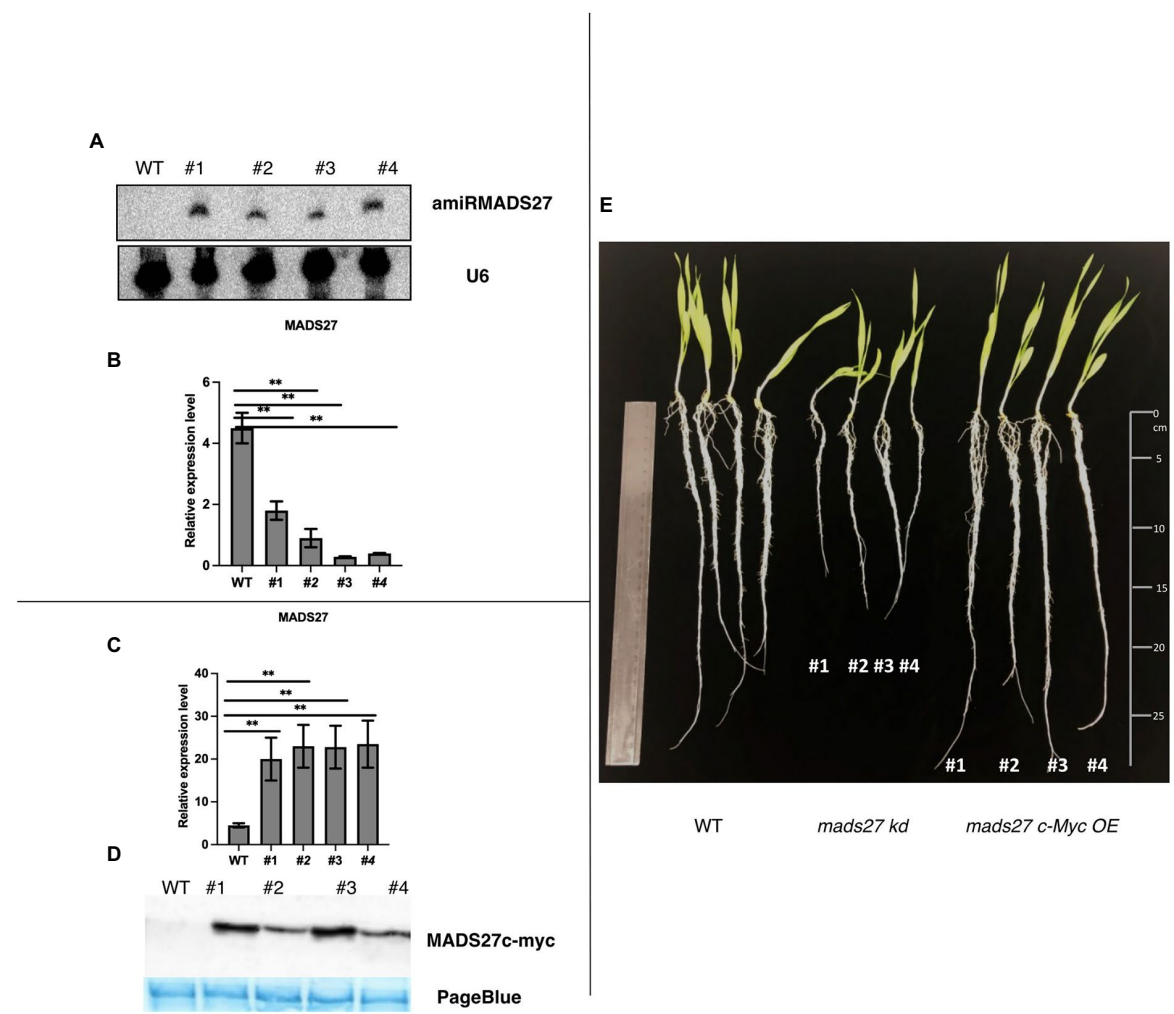
Characterization of *hvmads27* knockdown and overexpressing transgenic plants. **(A)** Northern blot analysis depicting the presence of amiRNAMADS27 in *mads27 kd* mutant lines using material from WT roots as control and U6 snRNA as loading control. **(B)** RT-qPCR analysis showing *HvMADS27* mRNA expression in *mads27 kd* mutant lines compared with WT plants relative to *ARF 1* mRNA level (Gene Bank: AJ508228.2), *x*-axis represents the transgenic lines and *y*-axis represents relative expression of mRNA. **(C)** RT-qPCR analysis showing *HvMADS27* mRNA expression in transgenic lines overexpressing *HvMADS27* with c-Myc tag relative to *ARF 1* mRNA level (Gene Bank: AJ508228.2); *x*-axis represents the transgenic lines and *y*-axis represents relative expression of *HvMADS27* mRNA. **(D)** Western blot experiment showing expression of HvMADS27 protein with c-Myc tag; lower panel showing loading control—PageBlue staining **(E)** 2-week old barley plants: WT plant, *mads27 kd*, and *mads27 c-Myc OE* mutants, respectively; *mads27 kd* plants exhibited short roots compared with the other transgenic lines. **(A–E)** #1, #2, #3, and #4 represent *mads27 kd*, *mads27 c-Myc OE* plants from four independent transgenic lines, respectively. *p* value was based on standard Student’s *t*-test **p < 0.01.

To elucidate the function of *HvMADS27* gene, we generated *HvMADS27* knockdown (*mads27 kd*) and overexpressing (*mads27 c-Myc OE*) transgenic barley plants by *Agrobacterium*-mediated immature embryo transformation. Knockdown plants were obtained using artificial microRNA targeting *HvMADS27* mRNA (amiRMADS27), and plants overexpressing *HvMADS27* contained c-Myc tag (Figures 2A–D; Supplementary Figures S5A–C, S6A,B). Four independent transgenic lines showing similar expression of *amiRMADS27* for *mads27 kd* lines and *HvMADS27* with c-Myc for *mads27 c-Myc OE* lines were selected for further experiments. RT-qPCR showed that there was a significant downregulation in the expression of *HvMADS27* mRNA in transgenic barley plants expressing amiRMADS27 (Figures 2A,B). In contrast, *HvMADS27* expression was significantly upregulated at the mRNA level in transgenic plants overexpressing *HvMADS27c-Myc*; moreover, HvMADS27 c-Myc protein was detected in all transgenic plants (Figures 2C,D). Phenotypic analysis of the plants showed that *hvmads27 kd* plants had shorter roots compared with WT plants, whereas the total root length of *mads27 c-Myc OE* plants was not significantly different from that of WT plants (Figure 2E). Hence, we incorporated different root categories into analysis and we measured separately seminal and lateral roots. It turned out that in the case of all *mads27 c-Myc OE* mutant lines, seminal roots were longer while lateral roots were shorter in comparison with WT plants (Supplementary Figure S7). However, upon N excess stress seminal roots in WT and *mads27 c-Myc OE* mutant plants are shorter and similar in length as in WT plants.

### High N stress induces root shortening in WT and *hvmads27oe* mutant plants while the root system of plants with knockdown of *HvMADS27* is not responsive to N excess

**Figure 3:**
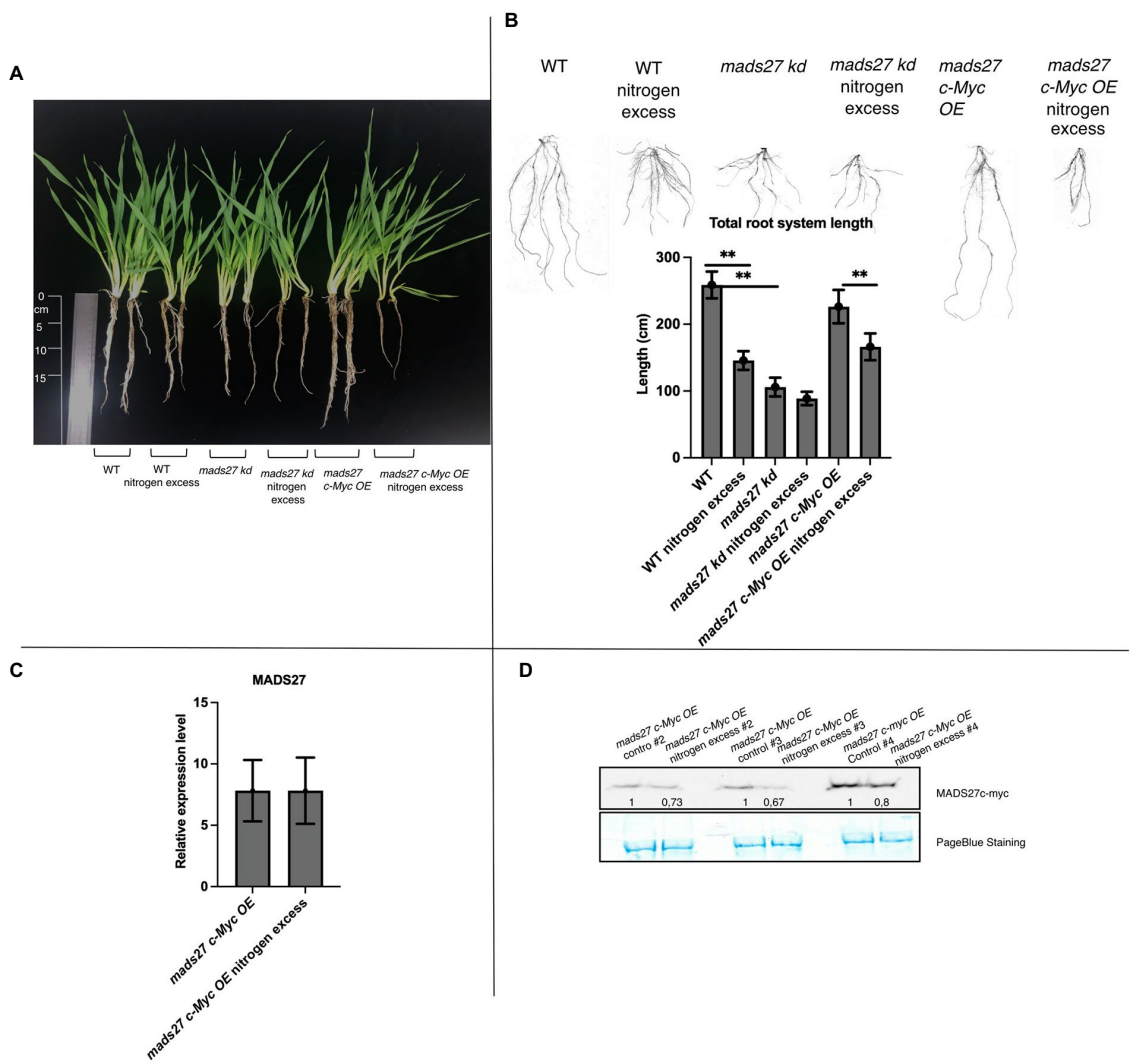
HvMADS27 regulates barley root architecture and excess N response. **(A)** WT and transgenic barley plants under control and excess N stress conditions. WT and *mads27 c-Myc OE* plants exhibited shorter roots under excess N condition compared with normal condition, whereas *mads27 kd* mutants exhibited shorter roots under control conditions, with no significant change under excess N stress. **(B)** Root length analysis using WinRHIZO software, with *x*-axis representing analyzed variants and *y*-axis representing total root length in cm. Scans of the root systems are provided. **(C)** RT-qPCR results depicting *HvMADS27* mRNA relative to *ARF 1* mRNA level (GenBank: AJ508228.2) expression in *mads27 c-Myc OE* mutant lines under control and excess N stress conditions. **(D)** Western blot analysis showed downregulation in HvMADS27 c-Myc protein level in *mads27 c-Myc OE* mutants under excess N treatment. PageBlue staining was used as loading control and numbers under MADS c-Myc signal depict results of densitometric analysis. Kruskal–Wallis test was performed to determine differences in root length; **p < 0.01.

Furthermore, the effect of excess N stress on the roots of WT, *hvmads27 kd*, and *mads27 c-Myc OE* plants were examined. The results showed that the root length of WT and *mads27 c-Myc OE* plants were significantly shorter under excess N stress than under normal condition. Additionally, *hvmads27 kd* mutants displayed significantly shorter roots under control conditions and did not respond to high N treatment (Figures 3A,B). Since it has been confirmed that the decrease in the root length under high N stress is associated with downregulation of *HvMADS27* mRNA, the response of *mads27 c-Myc OE* plants to high soil N and normal conditions was examined at both the transcript and protein levels (Figures 3C,D). Although excess N stress did not significantly affect the expression of *HvMADS27* in *mads27 c-Myc OE* lines at the mRNA level (Figure 3C), there was decrease in HvMADS27 protein level under excess N condition compared with normal condition (Figure 3D). This is in agreement with the observed downregulation in *HvMADS27* expression in WT plants at the mRNA level and most probably at the protein level as well. Downregulation of the HvMADS27 c-Myc protein in transgenic plants explains the similar behavior of their roots systems in excess N stress to WT plants.

### HvMADS27 TF regulates ABA level in barley roots by repressing *HvBG1* β-glucosidase transcription

**Figure 4:**
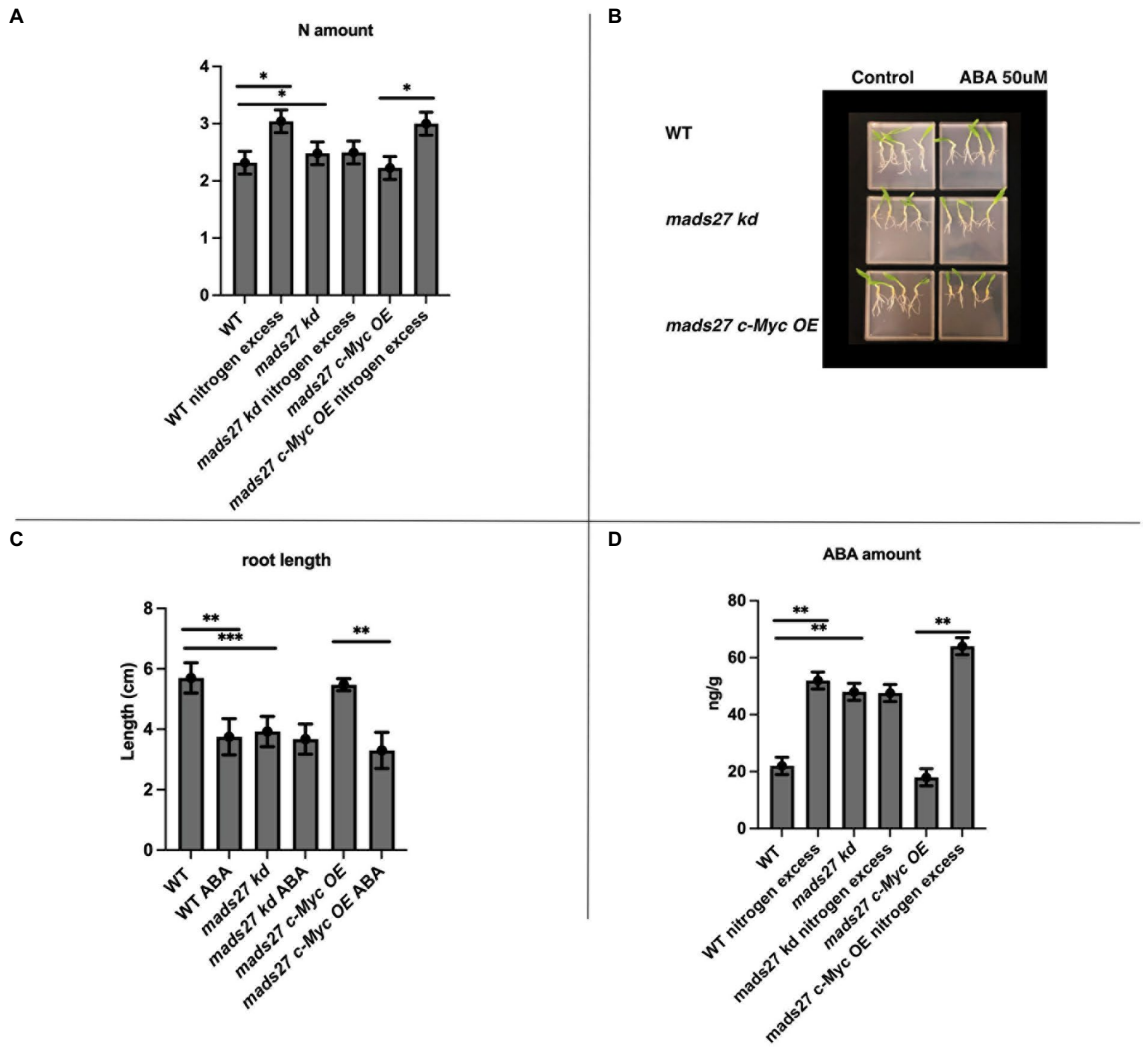
HvMADS27 controls N excess-dependent abscisic acid (ABA) accumulation in barley roots. **(A)** Determination of N content of the roots of WT*, mads27 kd*, and *mads27 c-Myc OE* plants using elemental analyzer. **(B)** ABA-sensitivity assay of the roots of WT and transgenic plants grown on ½ MS plates under control conditions or with 50 μM ABA supplementation. **(C)** Calculation of root length, with *x*-axis representing the variants and *y*-axis representing root length in cm. Roots of *mads27 kd* plants were shorter than those of plants from the other lines under control conditions and did not respond to ABA supplementation, whereas the roots of *mads27 c-Myc OE* mutants responded to ABA in WT-like manner. **(D)** ABA concentration of the roots was determined using ultra-high-pressure liquid chromatography, with the *x*-axis representing the variants and the *y*-axis representing ABA concentration in ng/g. There was an accumulation in ABA and N concentration in the roots of WT and *mads27 c-Myc OE* plants under excess N stress, whereas the accumulation of N and ABA in the roots of *mads27 kd* mutants was high both under control and excess N stress conditions. Mean comparison was performed using Student’s *t*-test; *p < 0.05, **p < 0.01, ***p < 0.0001.

In the present study, the molecular basis of the observed root phenotypes was investigated. First, the relationship between high N concentration in the rhizosphere and the root N content was examined. Overall, high rhizosphere N content caused accumulation of N in WT and *mads27 c-Myc OE* roots; however, higher amount of N was observed in *hvmads27 kd* plants under control condition, and the concentration was not significantly affected under high N stress (Figure 4A). The results correlate with the expression pattern of nitrogen sensor and transporter *NRT1.1* mRNA, which is upregulated upon excess N stress in roots of WT and *mads27 c-Myc OE* plants and exhibits high expression in roots of *hvmads27 kd* both in control conditions and N excess treatment (Supplementary Figure S4). Roots of *mads27 kd* plants in control conditions display similar characteristics to those of WT plants under excess N stress, but lower N concentration compared with that of WT roots subjected to high N stress. A plausible explanation for this could be that HvMADS27 TF may also regulate other nitrogen transporters active in barley roots. Moreover, we observed slightly reduced expression of *NRT1.1* in *mads27 kd* mutants; however, this change was not statistically significant (Supplementary Figure S4).

To investigate the mechanism of high N-dependent root shortening, we examined the possibility of ABA and N crosstalk in root system architecture, which has been previously reported in Arabidopsis and wheat (Finkelstein et al., 2002; Cutler et al., 2010; Yan et al., 2014; Sah et al., 2016; Harris and Ondzighi-Assoume, 2017; Jiao et al., 2020). First, the sensitivity to ABA in the roots of the barley mutants and WT plants was examined. The ABA sensitivity assay showed that there was a decrease in the root length of WT and *mads27 c-Myc OE* plants when exogenous ABA was supplied; however, the roots of *hvmads27 kd* mutants were short and not responsive to ABA treatment (Figures 4B,C). This result suggests that the HvMADS27 TF-mediated regulation of root growth is ABA-dependent. Therefore, the ABA content of the roots of WT plants and mutants was evaluated. Overall, there was an increase in the ABA content of the root of WT plants under excess N condition. However, the high ABA content of *hvmads27 kd* plants under control condition was not significantly affected by high soil N level. Plants overexpressing *HvMADS27* exhibited behavior similar to that of the WT (Figures 4C,D). Since the same observations concerning root shortening were made in WT and *hvmads27 kd, mads27 c-Myc OE* mutant plants subjected to N excess stress, this result confirmed the dominant role of HvMADS27 TF in controlling the ABA content in plant roots.

**Figure 5:**
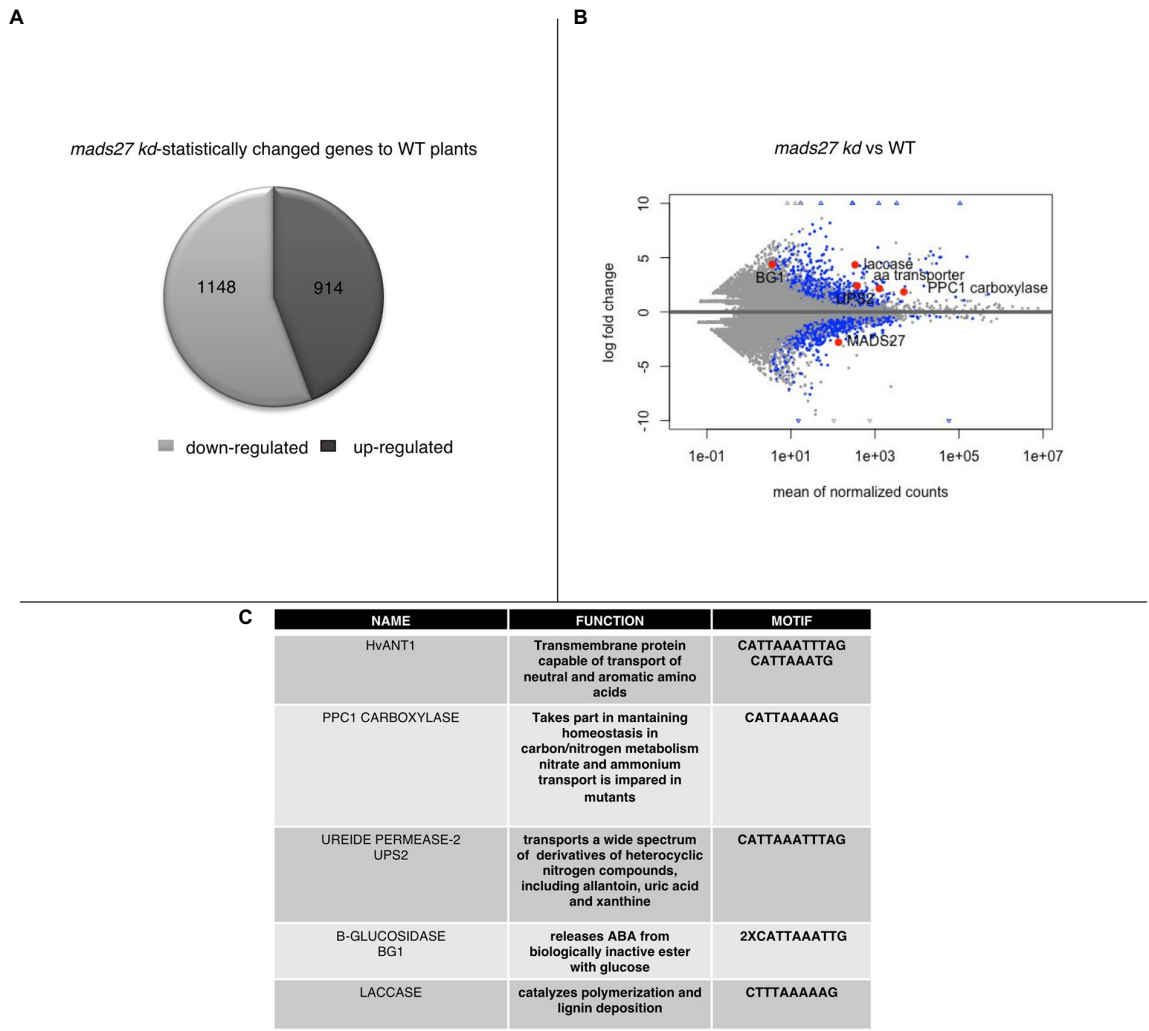
Transcriptomic analysis of roots of *mads27 kd* plants. **(A)** Pie chart depicting differentially expressed genes in the roots of *mads27 kd* mutants compared with that of WT roots. A total of 1,148 downregulated genes and 914 upregulated genes were identified. From upregulated group, genes harboring CArG motif in their promoter regions were selected for further analysis. **(B)** Volcano plot representing the expression level of candidate genes in the roots of *mads27 kd* mutant depicted in red dots. *X*-axis shows normalized counts and *y*-axis represents gene expression fold change. Blue dots in volcano plot represent significantly affected genes based on *p* value of Bonferroni correction. **(C)** List of the genes, function, and sequence of motif embedded in their promoters are shown in Table.

To better understand the mechanism of the observed behavior of *HvMADS27* mutants under high N stress, candidate genes regulated by HvMADS27 TF were identified by analysis of root transcriptome in WT and *hvmads27 kd* plants. We analyzed RNA-seq data for WT plants in control and N excess stress and identified 1,188 upregulated and 1817 downregulated genes (Supplementary Figure S8; Supplementary Table S3). Moreover, we identified 1,148 genes that were downregulated and 914 that were upregulated in *hvmads27 kd* plants when compared to WT plants in control conditions (Figure 5A). MADS-box transcription factors typically display inhibitory effects on regulated gene transcription; therefore, we mainly focused on the pool of upregulated genes and looked for the candidates that harbored in their promoters, the recognition motif for MADS-box TFs (CArG) binding (Guo et al., 2013; Muino et al., 2014; Aerts et al., 2018). Five potential candidate genes were selected (Figures 5B,C). *HvANT1* (HORVU6Hr1G034900) encoding a transmembrane protein involved in the transport of neutral and aromatic amino acids (Chen et al., 2001), *HvPPC1 CARBOXYLASE* (HORVU5Hr1G055350) encoding a protein involved in maintaining homeostasis in carbon/nitrogen metabolism (Li et al., 2020), *HvUPS2* (*UREIDE PERMEASE-2*; HORVU7Hr1G108790) encoding a transporter of nitrogen compounds (Rentsch et al., 2007), *HvLACCASE* (HORVU3Hr1G097860) encoding an enzyme that catalyzes the polymerization of lignins (Rittstieg et al., 2002), and *HvBG1* (*Β-GLUCOSIDASE*; HORVU2Hr1G023590) encoding an enzyme that releases ABA from ABA-glucose conjugate (Harris and Ondzighi-Assoume, 2017; Figure 5C; Supplementary Figure S9). Additionally, we considered the *NRT1.1* gene that was used as a nitrogen sensor because of the CArG motifs identified on its promoter (Supplementary Figure S9).

**Figure 6:**
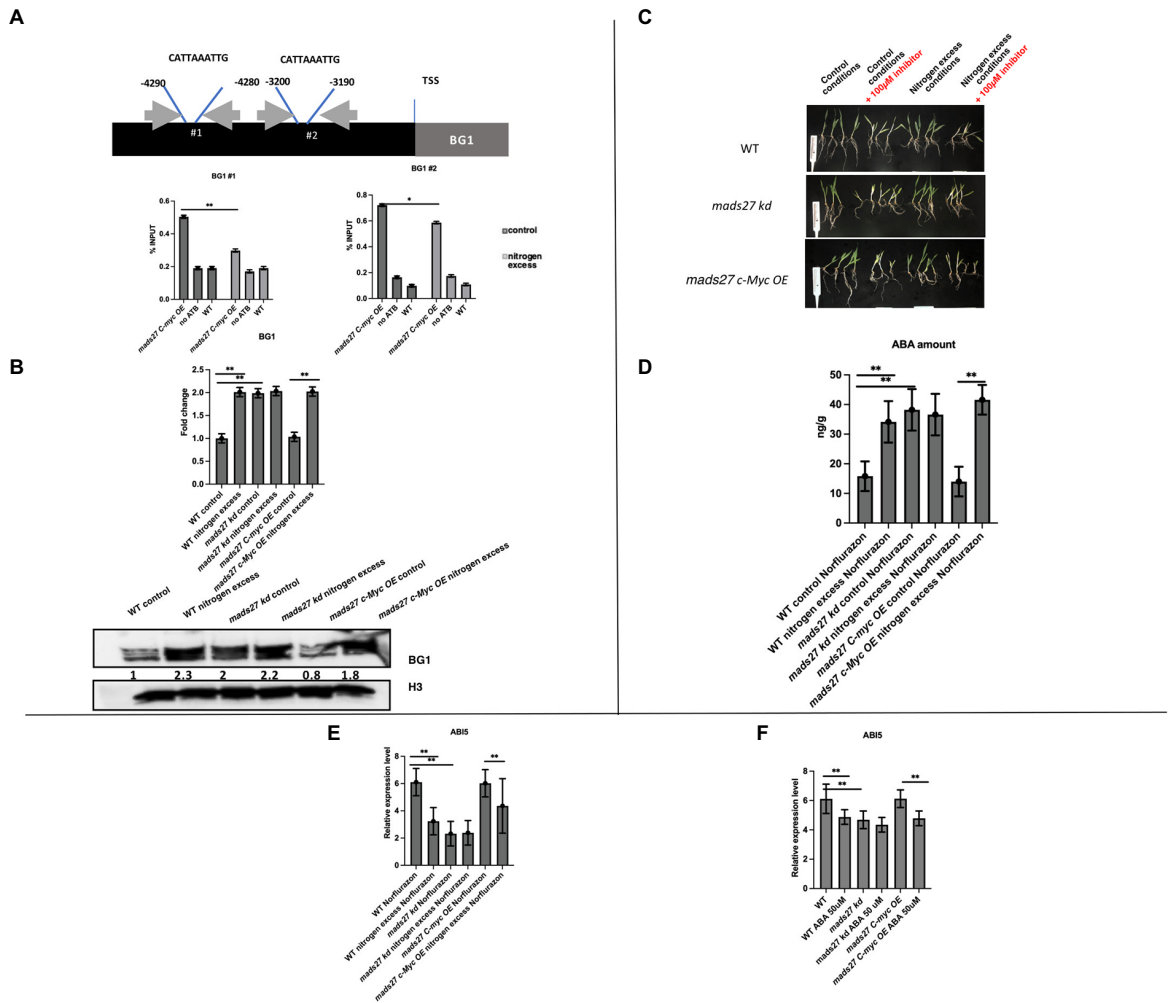
HvMADS27 transcription factor regulates expression of *BG1 β-GLUCOSIDASE* in barley roots. **(A)** Upper panel shows position of CArG motifs in *HvBG1* promoter, gray box indicates gene body, while black box indicates promoter sequence, blue lines flank the position of CArG motifs. Exact position of motif was identified by counting the number of nucleotides downstream of transcription start site. Gray arrows indicate the location of primers flanking CArG motifs. Lower panel presents the results of ChIP RT-qPCR of the roots of *mads27 c-Myc OE* plants under control and excess N stress conditions is presented using a graph. Accumulation of HvMADS27 on *HvBG1* promoter both in control and excess N conditions with lower occupation observed in stress. **(B)** Upper panel shows RT-qPCR results. There was an increase in the expression of *HvBG1* mRNA in the roots of WT and *mads27 c-Myc OE* lines under excess N stress, whereas *HvBG1* mRNA expression was high in the roots of *mads27 kd* mutant under control conditions and was not significantly affected by excess N stress. Lower panel shows western blot analysis of HvBG1 protein level in WT and mutant lines under control and excess N conditions. There was an increase in HvBG1 protein expression in the roots of *mads27 kd* mutant under both control and excess N conditions, whereas HvBG1 protein expression was increased in the roots of *mads27 c-Myc OE* mutant only under excess N stress. Below HvBG1 signal densitometric analysis is provided. HvBG1 level was validated using HvBG1 signal from roots of WT plants grown under control conditions. Signal from histon HvH3 was used as a loading control. **(C)** WT, *mads27 kd*, and *mads27 c-Myc OE* mutants plants grown under control and excess N stress conditions (supplemented with Norflurazon). Plants from *mads27 kd #3* line and *mads27 c-Myc OE #4* were used for the experiment. **(D)** The ABA content of the roots of WT and *mads27 kd, mads27 c-Myc OE* lines grown under control and excess N conditions (supplemented with Norflurazon) was determined by high-pressure liquid chromatography technique. There was an increase in the ABA concentration of Norflurazon-treated roots of WT, *mads27 kd*, and *mads27 c-Myc OE* plants under excess N stress, while in the case of *mads27 kd* ABA was high both under control and excess N stress conditions. **(E)** RT-qPCR results showing expression profiles of *HvABI5* mRNA in WT, *mads27 kd*, and *mads27 c-Myc OE* lines under control and excess N stress conditions supplemented with Norflurazon. *HvABI5* mRNA expression was significantly downregulated in the roots of WT and *mads27 c-Myc OE* lines under excess N, whereas the *HvAB15* mRNA was significantly lower in the root of *mads27 kd* mutants under both control and excess N stress conditions. **(F)** RT-qPCR results showing *HvABI5* mRNA level in WT and analyzed mutants in control and N excess conditions. Expression of *HvABI5* is downregulated upon ABA stress when compared to control conditions in WT plants, *mads27 c-Myc OE* plants. In *mads27 kd* plants the level of *HvABI5* is not changed upon the stress. **(E,F)** *x*-axis presents analyzed variants and *y*-axis expression level relative to *ARF 1* mRNA. **(A,B,D–F)** Statistical significance based on Student’s *t*-test, *p < 0.05, **p < 0.01.

To prove that HvMADS27 TF binds to the promoter regions of selected genes, we performed a ChIP-qPCR experiment using *mads27 c-Myc OE* lines and primers flanking CArG motifs in the promoters of all six genes, including the *NRT1.1* gene (Figure 6A; Supplementary Figure S9). Root material from WT plants treated with anti-c-Myc antibody and mutant root material to which we did not add antibody against the c-Myc tag were used as the control group during immunoprecipitation. Only in the case of the *HvBG1 Β-GLUCOSIDASE* gene, we observed enrichment of the PCR products representing fragments of the *HvBG1* promoter region containing the CArG motif. Moreover, HvMADS27 c-Myc occupancy on the *HvBG1* promoter decreased after high N treatment (Figure 6A). RT-qPCR analysis showed that there was an increase in *HvBG1* mRNA expression in the roots of WT and *hvmads27 c-Myc OE* mutant plants under nitrogen stress, while its level in *mads27 kd* plants was high under control conditions and remained unaffected under nitrogen stress (Figure 6B). Accordingly, western blot analysis of the HvBG1 protein level in roots of WT, *mads27 kd*, and *mads27 c-Myc OE* mutant plants under control conditions and excess N condition showed that HvBG1 was upregulated in the roots of WT and *mads27 c-Myc OE* plants under excess N condition. Regarding *hvmads27 kd* plants, the expression level of *HvBG1* was high both under control condition or under high N condition, with only slight upregulation after stress application (Figure 6B). These results indicated that HvMADS27 TF acts as a transcriptional repressor, at least in the case of *HvBG1*.

**Figure 7:**
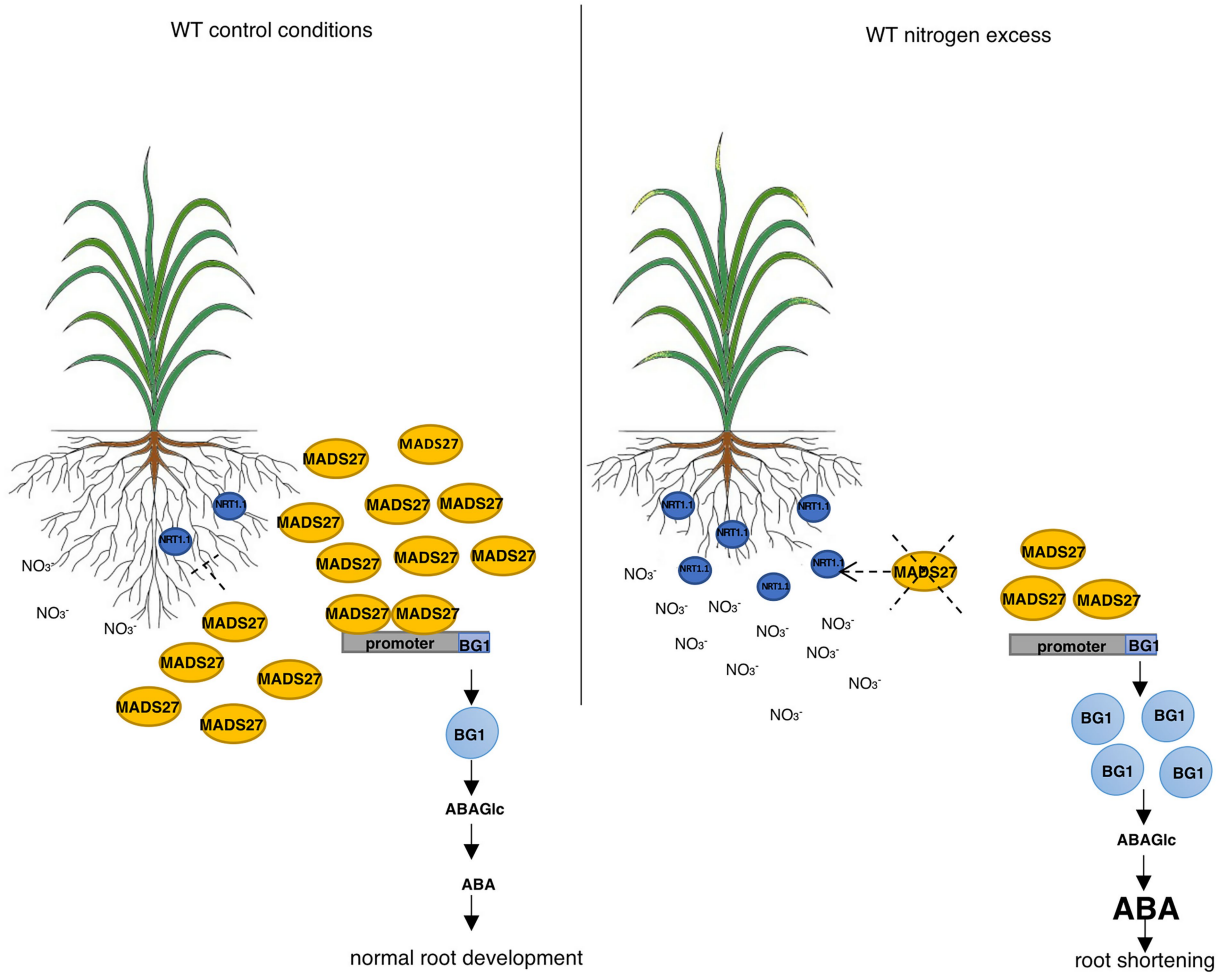
HvMADS27 regulates barley roots response to excess N stress. HvMADS27 regulated barley root response under excess N stress. Increased N concentration in the rhizosphere activates N sensor and transporter NRT1.1, which provides N influx to the root cells. High N concentration downregulates the expression of *HvMADS27*, which relieves the inhibition of *HvBG1* glucosidase expression. HvBG1 is activated and catalyzes the release of ABA from the ABA-glucose conjugate, which causes an increase in ABA levels and shortening of the barley roots.

Finally, to determine the relationship between the ABA content of WT, *mads27 c-Myc OE*, and *hvmads27 kd* plants and HvBG1 activity and ABA release from ABA-glucose conjugate, the ABA content of the roots of plants growing in the presence of the ABA biosynthesis inhibitor norflurazon were measured (Figures 6C,D; Dong et al., 2013). Plants treated with norflurazon displayed typical hypocotyl bleaching (Figure 6C; Salome, 2019). Additionally, there was an increase in the ABA content of WT and *mads27 c-Myc OE* mutant plants under high N content. Moreover, we observed high amounts of ABA in Norflurazon-treated roots of *mads27 kd* mutants under both control conditions and high N stress (Figure 6D). These results indicated that the increased amount of ABA in WT and *mads27 c-Myc OE* plants resulted from the mobilization of the ABA-glucose conjugate. Furthermore, the expression levels of *HvSCR*, *HvABI4*, and *HvABI5* transcription factor mRNAs, which are involved in root shortening in Arabidopsis, were examined (Harris, 2015; Ondzighi-Assoume et al., 2016; Figures 6E,F; Supplementary Figure S10). There was a decrease in the expression of *HvABI5* mRNA under high nitrogen stress in WT and *hvmads27 c-Myc OE* plants compared with *HvABI5* mRNA level in plants grown in control conditions. Regarding *hvmads27 kd* mutants *HvABI5* mRNA level was lower than in WT and unaffected under high N stress. *HvSCR* and *HvABI4* expression were unaffected in all analyzed plants and conditions (Supplementary Figures S10A,B). Taking all results together we propose the mechanism of HvMADS27-mediated regulation of *HvBG1* β-glucosidase that is N excess-dependent, where the presence of high amounts of N in the barley rhizosphere causes severe downregulation of *HvMADS27* expression and upregulation of *NRT1.1* expression. The decreased occupancy of HvMADS27 on the *HvBG1* promoter causes activation of this enzyme and release of ABA from its ester with glucose. The high levels of ABA in turn cause shortening of the plant roots (Figure 7).

## Discussion

In this study, we present a new mechanism of barley roots response to N excess stress: in control conditions barley HvMADS27 repressed the expression of *HvBG1* glucosidase and decreased the accumulation of active ABA in the roots. High N level caused a significant downregulation of HvMADS27, resulting in an increase in the expression of *HvBG1*, and consequently in an increased accumulation of ABA derived from the ABA-glucose conjugate.

In the presented study, there was a strong decrease in *HvMADS27* expression at the mRNA level in the root of barley under high N conditions. Phenotypic analyses of *HvMADS27* mutants showed that *HvMADS27* regulates barley root length in response to excess N stress. Plants with the knockdown of *HvMADS27* revealed shorter roots and did not shorten additionally in response to excess N stress. However, it was intriguing to observe similar effects on root length in the case of WT plant and plants overexpressing *HvMADS27 c-Myc*. In WT plants the level of HvMADS27 protein was controlled at the mRNA level, while it was not the case in *mads27 c-Myc OE* mutants where *HvMADS27* c-Myc cDNA was under control of 35S promoter. However, *HvMADS27 c-Myc* expression was indeed downregulated at the protein level in those plants in response to excess N stress. We were not able to measure directly the amount of HvMADS27 protein in WT plants regardless to control or stress conditions because antibodies specific to HvMADS27 are not available. Thus, we cannot compare the level of HvMADS27 protein in WT and *mads27 c-Myc OE* mutant plants. Similar effects of excess N on WT and *mads27 c-Myc OE* plants could be attributed to several plausible reasons. First, the interaction of HvMADS27 c-Myc and its partner was altered, which could affect the expression level of the partner, moreover a partner of HvMADS27 was affected by excess nitrogen, which could lead to degradation of HvMADS27 c-Myc. The effect of partner interaction on MADS-box TFs activity was previously reported in rice. It was evidenced by the EMSA assay that OsTB1 protein binds to OsMADS57 regulating its activity (Chen et al., 2018). Additionally, we cannot rule out the possibility that *HvMADS27 c-Myc* mRNA as well as HvMADS27 protein activities were lower than that of WT *HvMADS27* mRNA and protein. It has been shown that other MADS-box TFs may act as homo-or heterodimers or in larger complexes; therefore, a lack of stoichiometric balance between subunits of the complex in overexpressing lines could lead to the observed HvMADS27 c-Myc degradation (Hugouvieux and Zubieta, 2018).

Transcriptomic analysis of *hvmads27 kd* mutants showed that nitrate transporter *NRT1.1* gene expression was upregulated. The *NRT1.1* gene promoter contains MADS TF CArG-binding motifs. However, ChIP-qPCR experiments showed that HvMADS27 was not bound to the *NRT1.1* gene promoter. Moreover, *NRT1.1* mRNA expression was inversely correlated with *HvMADS27* expression level. Low *HvMADS27* expression under excess N stress was correlated with high levels of *NRT1.1* mRNA, indicating that HvMADS27 indirectly regulated *NRT1.1* expression.

Transcriptomic analysis of *hvmads27 kd* mutants also showed significant downregulation of HvMADS27 TF and a significant increase in *HvBG1* expression. Furthermore, ChIP-qPCR confirmed that the HvMADS27 transcription factor binds to the *HvBG1* gene promoter, which contained CArG motifs. Therefore, we conclude that the *HvBG1* glucosidase gene expression is repressed by HvMADS27 under control conditions, whereas downregulation of HvMADS27 under excess N stress induces *HvBG1* expression.

Wang et al. showed that wheat MADS-box TF, a homolog of Arabidopsis ANR1, activates expression of both TaBG1 and TaWabi5 - transcription factor that regulate the expression of N transporters from the TaNRT2 family (Wang et al., 2020). The nucleotide and amino acid sequences of TaANR1 and HvMADS27 in 97% similar to each other. However, in contrast to its wheat homolog, HvMADS27 is a repressor of *HvBG1* expression, as demonstrated by the upregulation of *HvBG1* expression in *hvmads27 kd* mutants. Additionally, there was an increase in *HvBGI* expression in WT plants under excess N condition at both mRNA and protein levels, which correlated with the downregulation of *HvMADS27* expression. The discrepancy between the modes of action of TaANR1 and HvMADS27 may be attributed to functional differentiation during the evolution of wheat and barley. Moreover, there are two *AtANR1* homologs in the wheat genome (AM502900.1 and XM_037629991.1), which is not the case in barley plants. This gene duplication may cause wheat AtANR1 MADS-box to create different complexes or dimerize with different partners than its barley counterpart, resulting in one being an activator and the other a repressor. Further experiments are needed to describe HvMADS27 and TaANR1 complexes. We also cannot exclude out the possibility that differences we observed in MADS27 behavior between wheat and barley result from different experimental approaches (N excess stress in barley vs. N starvation and supplementation to control conditions in wheat).

HvBG1 controls abscisic acid (ABA) levels in barley roots, with similar observations in wheat. ABA inhibits root growth through several mechanisms (Ondzighi-Assoume et al., 2016; Harris and Ondzighi-Assoume, 2017). In Arabidopsis, the *SCR* transcription factor gene expression is downregulated under higher N provision, with increase in ABA content, resulting in *ABI5* and *ABI4* expression upregulation, which could lead to root shortening. *ABI5* takes part in barley response to multiple abiotic stresses, especially drought which affects the movement and uptake of nutrients (Fahan et al., 2017; Collin et al., 2021). In this study, we observed a downregulation in *HvABI5* expression, which corresponds to root shortening in WT and *mads27 c-Myc OE* lines under excess N stress, and root shortening in *mads27kd* mutant plants under both control and excess N conditions. We performed analysis of *ABI5* mRNA level in control conditions and upon ABA treatment in WT and in all mutant plants. Indeed, the level of *ABI5* mRNA is downregulated in barley roots upon ABA treatment in WT plants and *hvMADS27 OE* mutant, while there is no significant change in *ABI5* mRNA level in *hvmads27 kd* mutant (Figure 6F). This confirms the difference between barley and Arabidopsis plants in response to ABA signaling. What is more, barley *ABI4* expression was not significantly downregulated (Supplementary Figure S10). Arabidopsis plants contain the *SCR* gene that is responsive to nitrogen; however, the expression of *SCR* in the roots of WT, *mads27 c-Myc OE*, and *mads27 kd* lines was not significantly affected by high N stress (Supplementary Figure S10). Thus, it seems that the mechanisms regulating *ABI5* expression in barley and Arabidopsis were different in response to N concentration. We also analyzed RNAseq data for the level of other core genes known to be involved in *de novo* biosynthesis of ABA and ABA signaling. The analysis was performed using RNAseq data from roots of *mads27 kd* plants compared to wild-type plants. Following genes were taken into consideration: *NCED1*, *NCED2*, *ABAOH8*, *ZEP, PP2C*, *PYR1*, *PYL2*, *PYL5*, *PYL8*, *ABI1*, *ABI2*, and *ABI3*. Generally, we observed no changes in their expression levels, which confirms that HvMADS27 is not involved in the control of ABA *de novo* biosynthesis but may be involved in ABA signaling (see Supplementary Table S3).

To identify important processes affecting N acquisition, root traits essential for N uptake and the expression of N uptake-related genes in the roots of barley were assessed. The root system of barley is a fibrous type consisting of seminal and nodal roots (crown roots) and lateral roots of diverse acquisitive capacity (Jia et al., 2019). Previous studies have shown that barley produces more lateral roots in response to N provision (Blaser et al., 2020). Similarly, there was an increase in lateral root formation in response to excess N stress in WT, *hvmads27 kd*, and *hvmads27 c-Myc OE* lines in the present study. Moreover, there was no significant difference in lateral root number of the WT and mutant lines under control conditions, indicating that *HvMADS27* regulates total root length but not lateral root number (Figure 3B; Supplementary Figure S11). Deeper rooting increases N uptake by increasing total soil exploration. Therefore, plants optimize roots to respond to changing mobile nutrients, such as nitrate, that tend to be more abundant in deeper soil layers (Giehl and von Wiren, 2014; Liu et al., 2020). Similar studies have been performed with *A. thaliana*: Excess N has been reported to shorten the root length and enhances the formation of lateral roots in Arabidopsis (Walch-Liu et al., 2006). Thus, this feature seems to be conserved across plant kingdom, although detail mechanisms underlying root shortening in N excess stress may differ between plant species.

Finally, *HvMADS27* mRNA, similar to several other MADS-box TF mRNAs, is specifically targeted by miRNA from the MIR444 family (Sunkar et al., 2005, 2008; Lu et al., 2008). Degradome analysis showed that miRNA444c cleaved *HvMADS27* mRNA under control conditions (Supplementary Figure S1). However, the expression of mature miRNA444c did not correlate with *HvMADS27* mRNA downregulation under high N condition. These findings suggest that miRNA444c did not significantly affect the expression of *HvMADS27* under these conditions. However, based on our analysis of expression patterns in barley developmental stages, miRNA444c-mediated regulation of *HvMADS27* expression may occur during other stages of plant development and/or in response to other stresses. Moreover, there were significant variations in miR444c level during week 1, 2, 3, and 6, and 68th day of barley growth, suggesting the role of miR444c and MADS27 TF during barley development (see Figure 1B).

The findings of the present study provide novel insights into barley root response to excess N stress and constitute a basis for further research on the mechanism of cereal response to over-fertilization. Moreover, the findings of the study provide candidate genes for the breeding and development of excess N-resistant crop varieties better adapted to leached agroecosystem soils. Moreover, this research creates new areas for future studies which can entail analyzing miRNA444c and *HvMADS27* roles in developmental stages that were not taken under consideration so far, exploring the structure and differences in partners that build complexes with HvMADS27 and its homologs in other crop species or exploring the role of ABI5 in barley root response to N excess stress.

## Data availability statement

The original contributions presented in the study are publicly available. This data can be found at: NCBI, PRJNA746688, https://dataview.ncbi.nlm.nih.gov/object/PRJNA746688?reviewer=85l4hm6uj4i81nrjfeihgp001s.

## Author contributions

AS performed the majority of experiments included in this work and wrote the manuscript draft. AP performed degradome analysis and participated in barley protoplast preparation and discussion. AG took part in the transgenic line construction. DB and WMK carried out bioinformatic analyses. MZ helped in the root architecture analysis and participated in the discussion. KS participated in the phenotypic analysis of root behavior. JD participated in CHIP experiments and discussion. MB performed nuclear localization experiments in plant protoplasts. PN participated in degradome library construction. JK participated in ABA measurements and discussion. MW and IR performed N amount analysis and participated in the discussion. WH participated in amiRNAMADS27 transgenic line construction. AJ contributed to the discussion and writing of the results. ZS-K designed experiments and wrote the manuscript. All authors contributed to the article and approved the submitted version.

## Funding

This study was funded by the OPUS project (2016/23/B/NZ9/00862) and PRELUDIUM project (2019/35/N/NZ9/01971) from the National Science Centre, Poland. Production of transgenic barley plants was possible thanks to Short Term Scientific Mission (STSM) founded by iPlanta Organization COST Action CA15223. Authors also received financial support from the Initiative of Excellence—Research University (05/IDUB/2019/94) at Adam Mickiewicz University, Poznan.

## Conflict of interest

Since 16/11/2021, the co-author AS has been employed by Frontiers Media SA. AS declared his/her affiliation with Frontiers, and the handling Editor states that the process nevertheless met the standards of a fair and objective review.

The remaining authors declare that the research was conducted in the absence of any commercial or financial relationships that could be construed as a potential conflict of interest.

## Publisher’s note

All claims expressed in this article are solely those of the authors and do not necessarily represent those of their affiliated organizations, or those of the publisher, the editors and the reviewers. Any product that may be evaluated in this article, or claim that may be made by its manufacturer, is not guaranteed or endorsed by the publisher.

## Supplementary material

The Supplementary material for this article can be found online at: https://www.frontiersin.org/articles/10.3389/fpls.2022.950796/full#supplementary-material

Supplementary Figure S1 HvMiRNA444c targets *HvMADS27* transcription factor mRNA. Degradome analysis of 68-day old barley plant grown under control conditions. Perfect complementarity between *HvMADS27* mRNA (accession number: HORVU2Hr1G080490.4) and miR444c is shown. The x-axis of the graph represents the length of the *HvMADS27* transcript (722 nt), and the y-axis depicts the abundance of identified cleavage and degradation fragments in read numbers. The red line indicates the miRNA444c- mediated cleavage site at position 287.

Supplementary Figure S2 *HvMADS27* is mainly expressed in roots of two-week old barley plants. Results of RT-PCR of organ samples of two-week old barley plants; expression of *ARF1* mRNA was used as a loading control.

Supplementary Figure S3 MADS27-GFP localizes in the nucleus. Transient co-expression of fluorescent proteins in **(A)** Arabidopsis protoplasts and **(B)** Barley protoplast. RFP-SERRATE was used as a nuclear marker.

Supplementary Figure S4 Plants growing under N scarcity and excess N conditions. **(A)** WT plants grown under high N stress condition. **(B)** Expression level of *NRT1.1* mRNA transporter using RT-qPCR technique. *NRT1.1* mRNA was upregulated in response to high N stress. X-axis presents analyzed variants and y-axis shows expression level relative to *ARF 1* mRNA. **(C)** WT plants growing in N deprivation conditions. **(D)** RT-qPCR results depicting expression level of *NRT1.1* mRNA under N scarcity conditions. *NRT1.1* mRNA was downregulated under N scarcity. **(E)** Expression of *NRT1.1* mRNA in WT, *mads27 kd*, and *mads27 c-Myc OE* mutants under control and high N conditions. *NRT1.1* mRNA was upregulated in *mads27 kd* mutant and its expression was not affected under high N stress. Excess N stress caused an upregulation in *NRT1.1* mRNA expression in *mads27 c-Myc OE* mutant lines. Above the graphs statistical significance is provided based on standard Student *t*-test, **p < 0.01.

Supplementary Figure S5 amiRMADS27 in miRNA167h precursor. **(A)** Structure of barley *MIR167h* gene with red bar marking mature miRNA167h position and blue bar marking miRNA^✶^. TSS-transcription start site. **(B)** Structure and sequence of pre-miRNA167h, with red color indicating mature miRNA167h sequence and blue color miRNA^✶^. Nucleotides marked in green were changed in amiRMADS27 precursor to improve hairpin structure. **(C)** Pre-miRNA167h with amiRMADS27 and its cognate miRNA^✶^. ΔG = Gibbs free energy value of particular pre-miRNA.

Supplementary Figure S6 Phenotypes of plants from four independent transgenic lines. **(A)** Plants from four independent transgenic barley lines with knock-down of (*mads27 kd* #1, *mads27 kd* #2, *mads27 kd* #3, and *mads27 kd* #4, respectively). **(B)** Plants from four independent transgenic barley lines overexpressing *HvMADS27* with c-Myc tag (*mads27 c-Myc OE* #1, *mads27 c-Myc OE* #2, *mads27 c-Myc OE* #3, and *mads27 c-Myc OE* #4, respectively).

Supplementary Figure S7 Analysis of length of seminal and lateral roots in WT, *mads27 kd* and *mads27 c-Myc OE* plants in control and N excess stress conditions. In the case of all *mads27 c-Myc OE* mutant lines, seminal roots are longer while lateral roots are shorter in comparison to WT plants in control conditions. Upon N excess stress seminal roots in WT and *mads27 c-Myc OE* mutant plants are shorter and similar in length as in WT plants. Different capital letters indicate significant difference between root length means among WT, *mads27 c-Myc OE*, and *mads27 kd* within a given nitrogen condition and the same letters indicate lack of statistical significance according to Dunn test. Error bars represent the standard error. value of *p* <0.05.

Supplementary Figure S8 RNA-seq data from roots of WT barley plants grown in control conditions and N excess stress. Volcano plot representing comparison between RNA-seq data from roots of WT plants grown in control and N excess stress. Red dots depict genes changed with statistical significance based on *p*-value of Bonferroni correction. X-axis shows normalized counts and y-axis represents gene expression fold change.

Supplementary Figure S9 ChIP-qPCR results of candidate genes for regulation by HvMADS27. **(A–E)** Depict the results of ChIP-qPCR for *HvANT1*, *PPC1*, *UPS1*, *LACCASE*, and *NRT1.1* promoter genes, respectively. Black boxes represent promoter sequences, grey boxes depict gene bodies, and blue lines flank the position of CArG motifs. The exact positions of motifs were provided by the numbers of nucleotides upstream of the transcription start site. Grey arrows show the location of primers flanking the CArG motifs. Graphs depict ChIP-qPCR results from roots of *mads27 c-Myc OE* plants in control and excess N stress, using primers flanking CArG motifs. We observe no accumulation of HvMADS27 on analyzed promoters. No Atb-ChIP qPCR with no added anti-myc antibody or WT wild-type plants

Supplementary Figure S10 Expression pattern of *HvSCR, HvABI4* genes. **(A)** RT-qPCR result showing *HvSCR* mRNA level in WT and mutants under control and high soil N conditions, where x-axis represents analyzed variants and y-axis represents expression level relative to *ARF1* mRNA. There was no significant change in *HvSCR* expression in the mutants compared with WT plant. **(B)** RT-qPCR results showing *HvABI4* mRNA level in WT and analyzed mutants in control and excess N conditions where x-axis presents analyzed variants and y-axis expression level relative to *ARF 1* mRNA. Expression of *HvABI4* is not changed in excess N stress in WT and mutants when compared to control conditions.

Supplementary Figure S11 Number of lateral roots in WT and *hvmads27* mutant plants. Number of lateral roots calculated based on scans of root systems of WT, *mads27 kd* and *mads27 c-Myc OE* mutants, where x-axis shows analyzed variants and y-axis number of observed lateral roots. In each case (WT, *mads27 kd*, *mads27 c-Myc OE*) number of lateral roots was higher under high N stress. Statistical significance based on standard Student *t*-test is provided, **p < 0,01.

## Footnotes

^1^ https://www.wmd3.weigelworld.org

^2^ https://www.jic.ac.uk/technologies/genomic-services/bract

^3^ https://plants.ensembl.org/Hordeum_vulgare/

^4^ https://mafft.cbrc.jp/alignment/

^5^ www.wmd3.weigelworld.org
