# Supplementary figures and images for: Excess nitrogen responsive HvMADS27 transcription factor controls barley root architecture by regulating abscisic acid level
Source: Front Plant Sci. 2022 Sep 12;13:950796. doi: 10.3389/fpls.2022.950796 (PMC9511987; doi:10.3389/fpls.2022.950796)

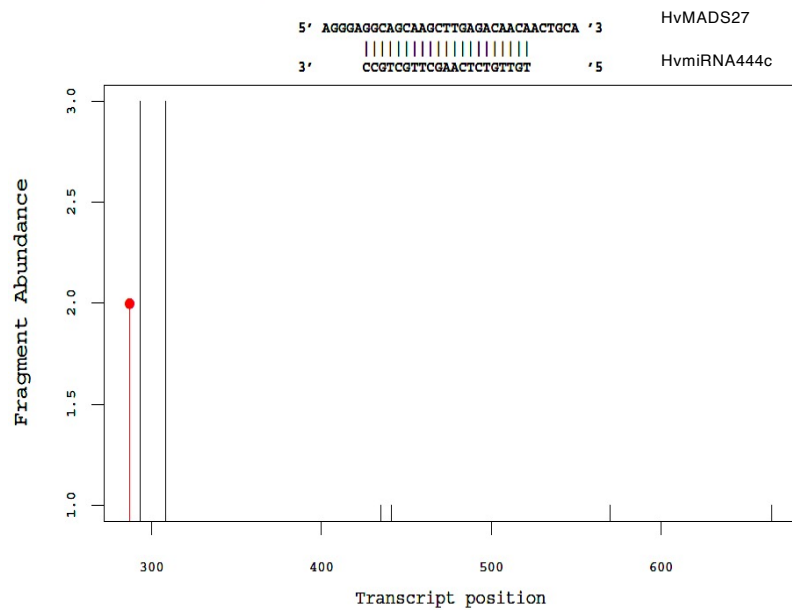

Supplement: Supplementary Figure S1 — HvMiRNA444c targets HvMADS27 transcription factor mRNA. Degradome analysis of 68-day old barley plant grown under control conditions. Perfect complementarity between HvMADS27 mRNA (accession number: HORVU2Hr1G080490.4) and miR444c is shown. The x-axis of the graph represents the length of the HvMADS27 transcript (722 nt), and the y-axis depicts the abundance of identified cleavage and degradation fragments in read numbers. The red line indicates the miRNA444c- mediated cleavage site at position 287. [file Data_Sheet_1.ZIP › Supplementary data/Figure S1.pdf]

A

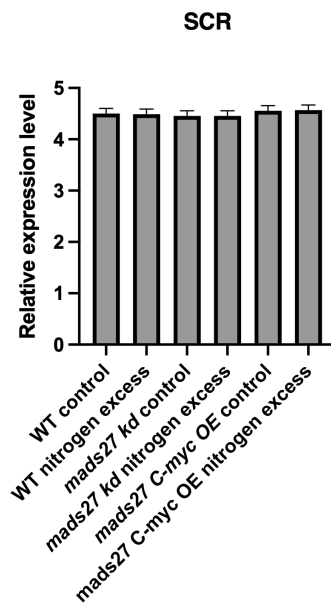

B

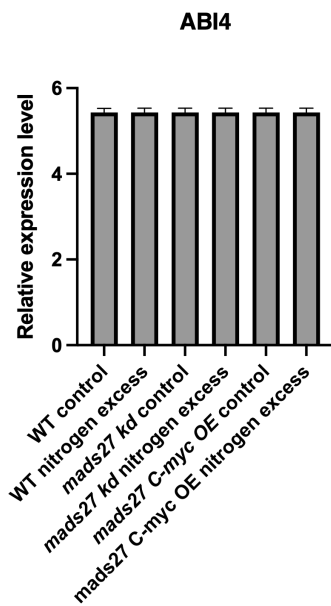

Supplement: Supplementary Figure S1 — HvMiRNA444c targets HvMADS27 transcription factor mRNA. Degradome analysis of 68-day old barley plant grown under control conditions. Perfect complementarity between HvMADS27 mRNA (accession number: HORVU2Hr1G080490.4) and miR444c is shown. The x-axis of the graph represents the length of the HvMADS27 transcript (722 nt), and the y-axis depicts the abundance of identified cleavage and degradation fragments in read numbers. The red line indicates the miRNA444c- mediated cleavage site at position 287. [file Data_Sheet_1.ZIP › Supplementary data/Figure S10.pdf]

A

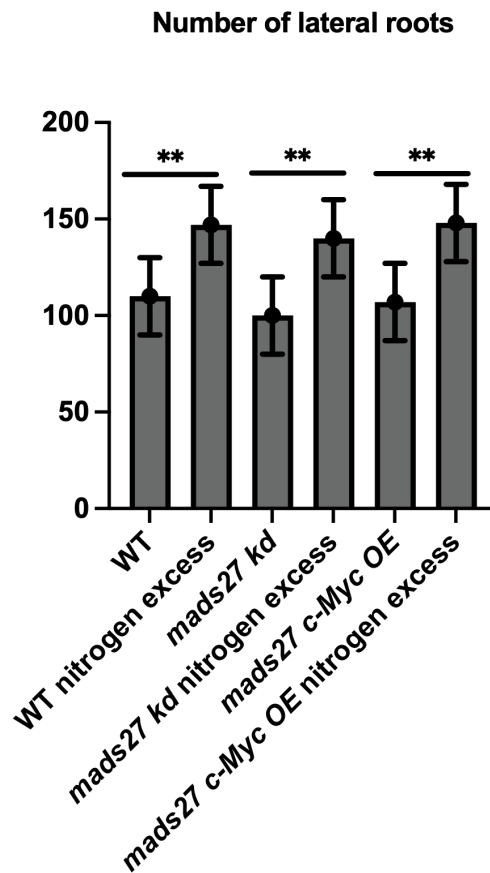

Supplement: Supplementary Figure S1 — HvMiRNA444c targets HvMADS27 transcription factor mRNA. Degradome analysis of 68-day old barley plant grown under control conditions. Perfect complementarity between HvMADS27 mRNA (accession number: HORVU2Hr1G080490.4) and miR444c is shown. The x-axis of the graph represents the length of the HvMADS27 transcript (722 nt), and the y-axis depicts the abundance of identified cleavage and degradation fragments in read numbers. The red line indicates the miRNA444c- mediated cleavage site at position 287. [file Data_Sheet_1.ZIP › Supplementary data/Figure S11.pdf]

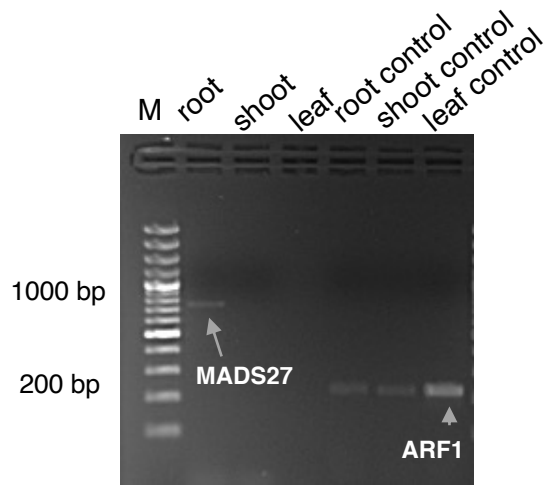

Supplement: Supplementary Figure S1 — HvMiRNA444c targets HvMADS27 transcription factor mRNA. Degradome analysis of 68-day old barley plant grown under control conditions. Perfect complementarity between HvMADS27 mRNA (accession number: HORVU2Hr1G080490.4) and miR444c is shown. The x-axis of the graph represents the length of the HvMADS27 transcript (722 nt), and the y-axis depicts the abundance of identified cleavage and degradation fragments in read numbers. The red line indicates the miRNA444c- mediated cleavage site at position 287. [file Data_Sheet_1.ZIP › Supplementary data/Figure S2.pdf]

A

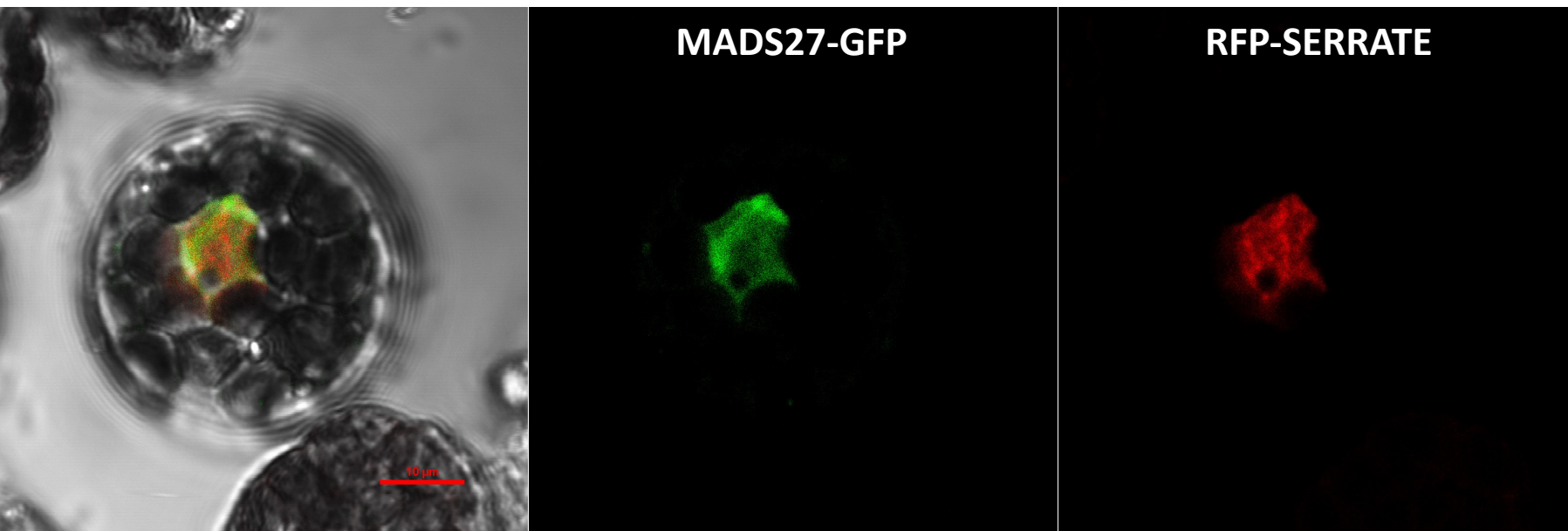

B

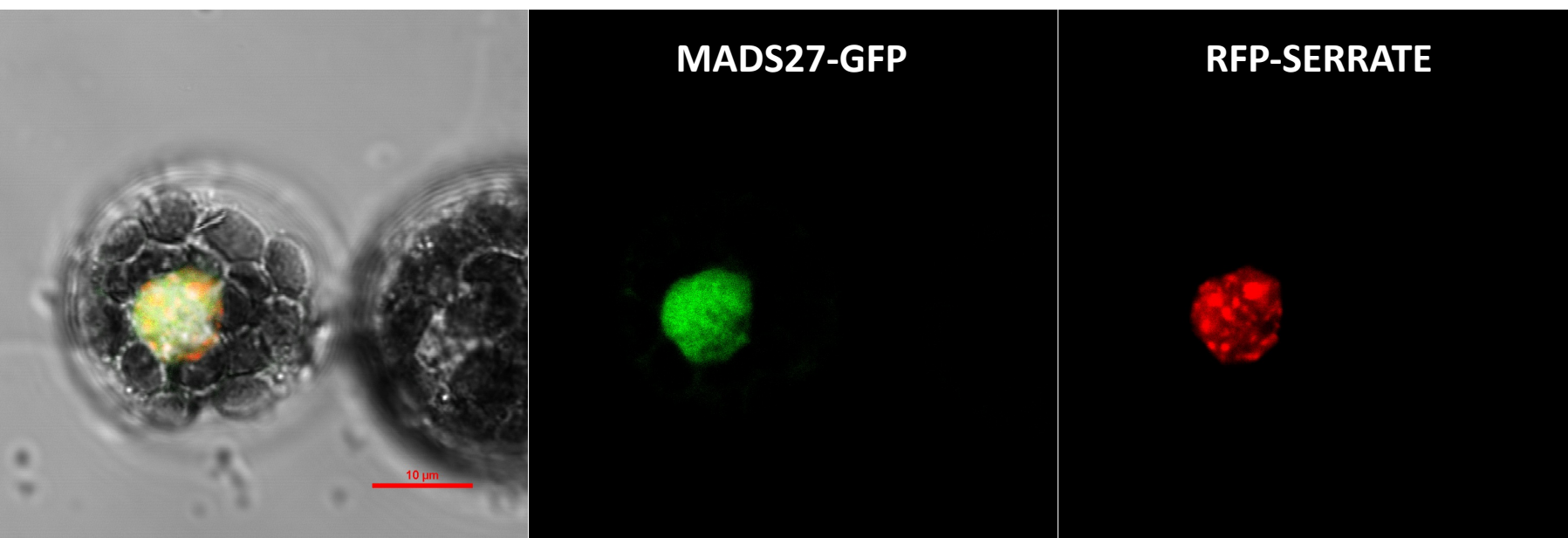

Supplement: Supplementary Figure S1 — HvMiRNA444c targets HvMADS27 transcription factor mRNA. Degradome analysis of 68-day old barley plant grown under control conditions. Perfect complementarity between HvMADS27 mRNA (accession number: HORVU2Hr1G080490.4) and miR444c is shown. The x-axis of the graph represents the length of the HvMADS27 transcript (722 nt), and the y-axis depicts the abundance of identified cleavage and degradation fragments in read numbers. The red line indicates the miRNA444c- mediated cleavage site at position 287. [file Data_Sheet_1.ZIP › Supplementary data/Figure S3.pdf]

A

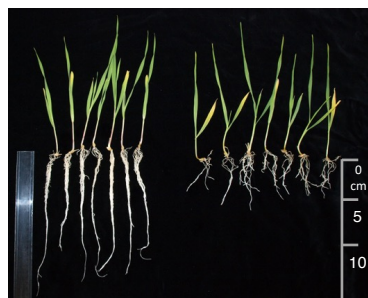

WT  
control

WT nitrogen  
excess

B

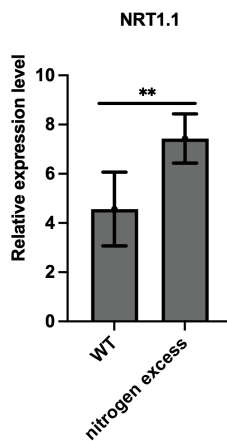

C

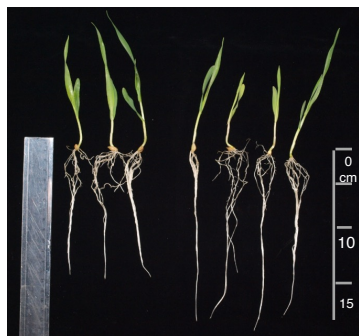

WT  
control

WT nitrogen  
deprivation

D

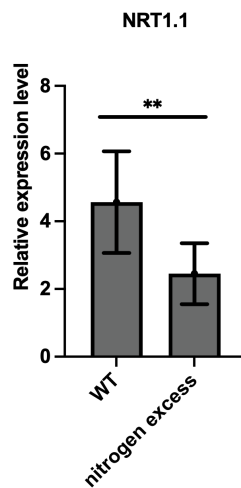

E

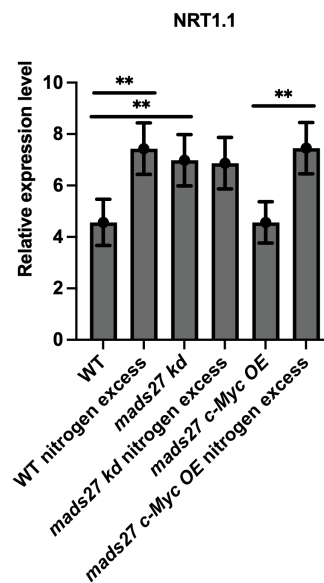

Supplement: Supplementary Figure S1 — HvMiRNA444c targets HvMADS27 transcription factor mRNA. Degradome analysis of 68-day old barley plant grown under control conditions. Perfect complementarity between HvMADS27 mRNA (accession number: HORVU2Hr1G080490.4) and miR444c is shown. The x-axis of the graph represents the length of the HvMADS27 transcript (722 nt), and the y-axis depicts the abundance of identified cleavage and degradation fragments in read numbers. The red line indicates the miRNA444c- mediated cleavage site at position 287. [file Data_Sheet_1.ZIP › Supplementary data/Figure S4.pdf]

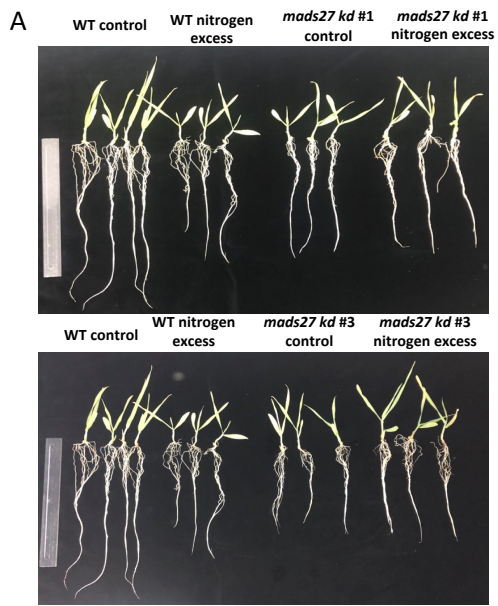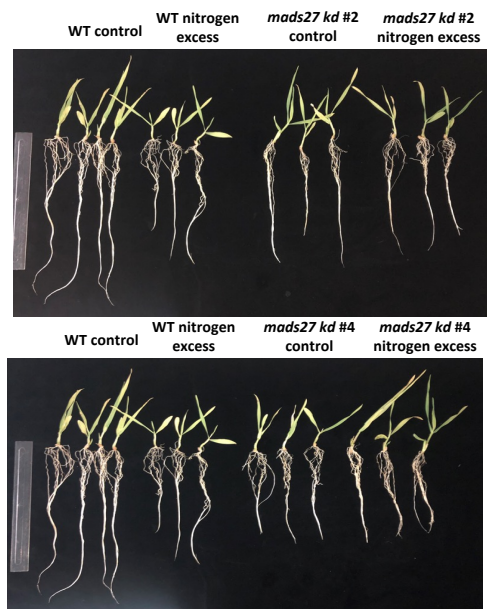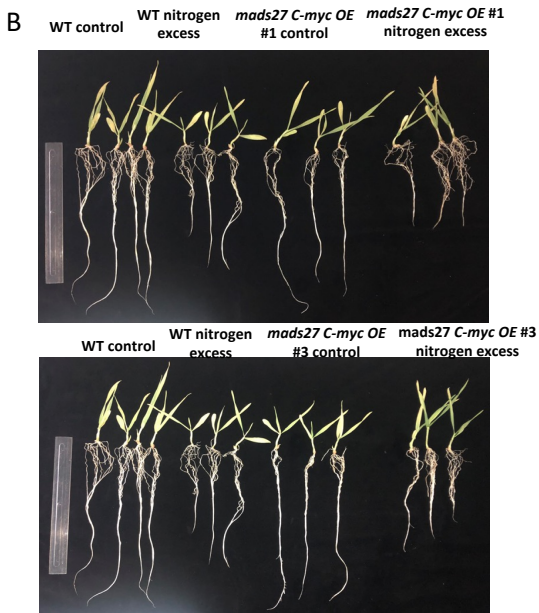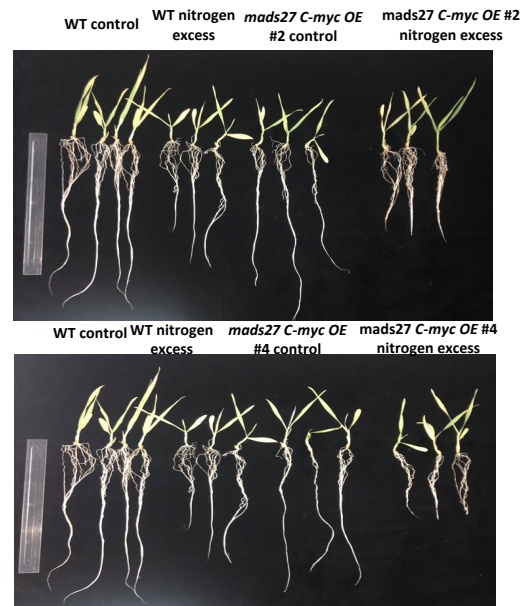

Supplement: Supplementary Figure S1 — HvMiRNA444c targets HvMADS27 transcription factor mRNA. Degradome analysis of 68-day old barley plant grown under control conditions. Perfect complementarity between HvMADS27 mRNA (accession number: HORVU2Hr1G080490.4) and miR444c is shown. The x-axis of the graph represents the length of the HvMADS27 transcript (722 nt), and the y-axis depicts the abundance of identified cleavage and degradation fragments in read numbers. The red line indicates the miRNA444c- mediated cleavage site at position 287. [file Data_Sheet_1.ZIP › Supplementary data/Figure S6.pdf]

A

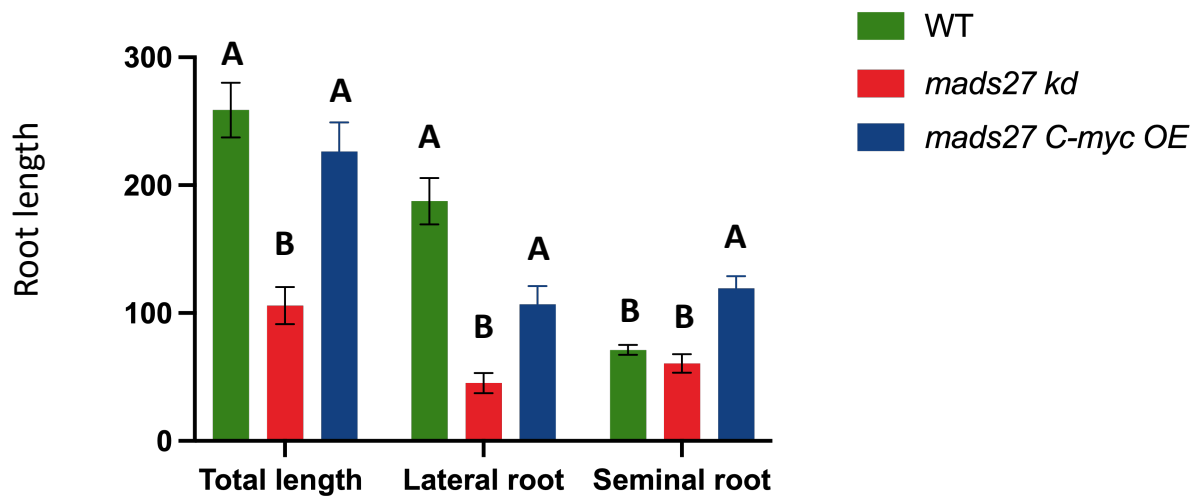

B

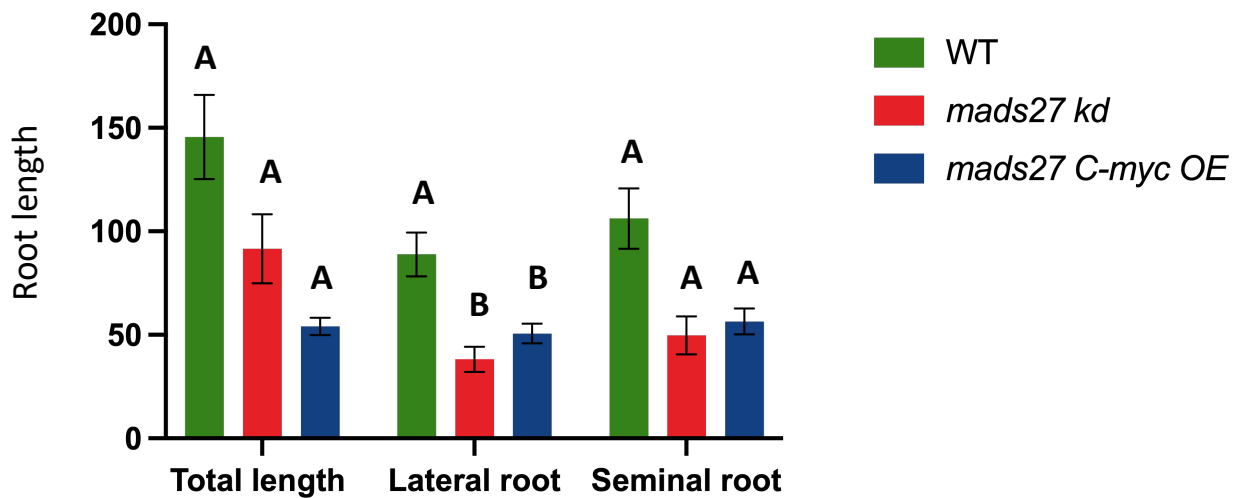

Supplement: Supplementary Figure S1 — HvMiRNA444c targets HvMADS27 transcription factor mRNA. Degradome analysis of 68-day old barley plant grown under control conditions. Perfect complementarity between HvMADS27 mRNA (accession number: HORVU2Hr1G080490.4) and miR444c is shown. The x-axis of the graph represents the length of the HvMADS27 transcript (722 nt), and the y-axis depicts the abundance of identified cleavage and degradation fragments in read numbers. The red line indicates the miRNA444c- mediated cleavage site at position 287. [file Data_Sheet_1.ZIP › Supplementary data/Figure S7.pdf]

## WT control vs excess N stress

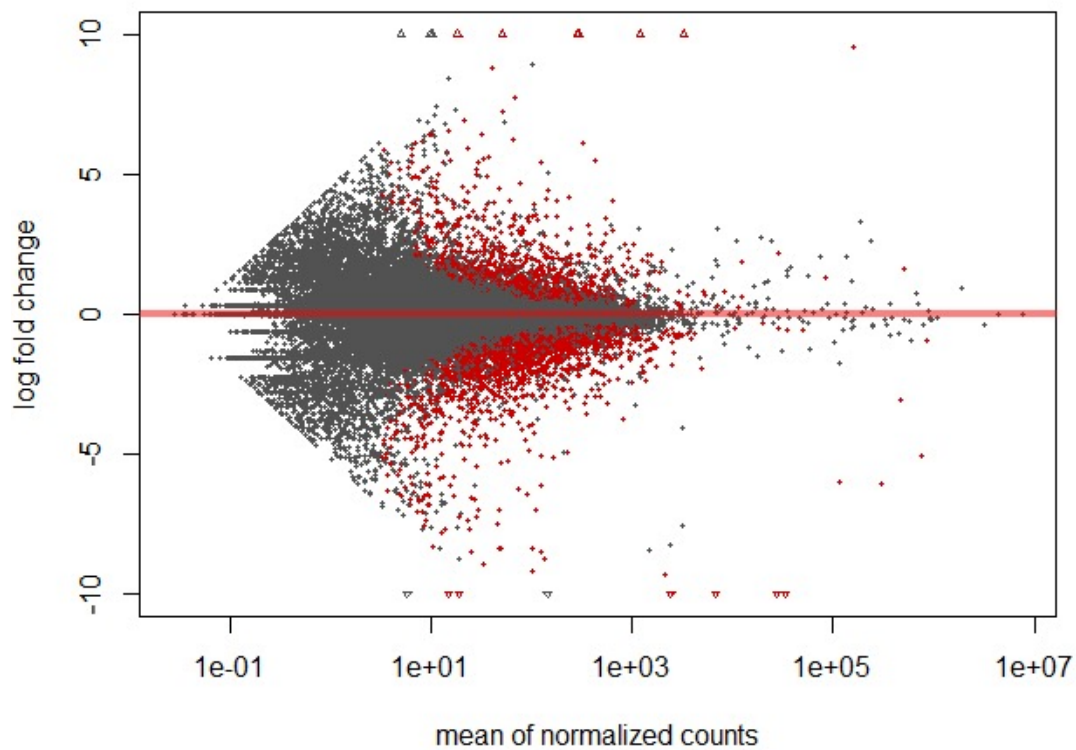

Supplement: Supplementary Figure S1 — HvMiRNA444c targets HvMADS27 transcription factor mRNA. Degradome analysis of 68-day old barley plant grown under control conditions. Perfect complementarity between HvMADS27 mRNA (accession number: HORVU2Hr1G080490.4) and miR444c is shown. The x-axis of the graph represents the length of the HvMADS27 transcript (722 nt), and the y-axis depicts the abundance of identified cleavage and degradation fragments in read numbers. The red line indicates the miRNA444c- mediated cleavage site at position 287. [file Data_Sheet_1.ZIP › Supplementary data/Figure S8.pdf]

A

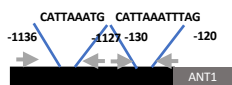

B

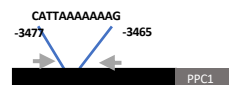

ANT1 #1

ANT1 #2

PPC1 CARBOXYLASE

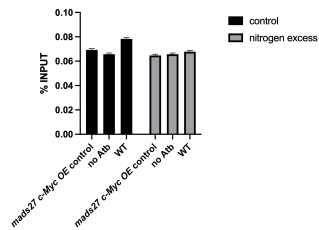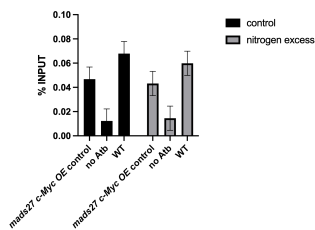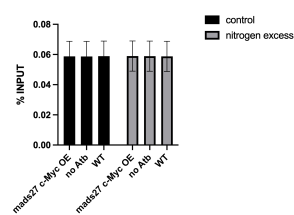

C

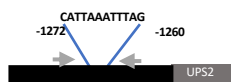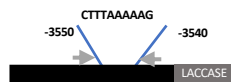

E

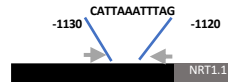

UPS2 UREIDE PERMEASE-2

LACCASE

NRT1.1

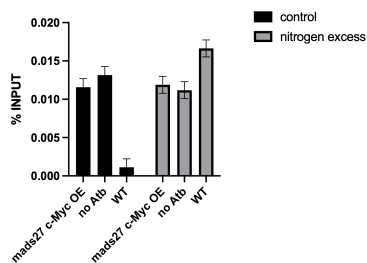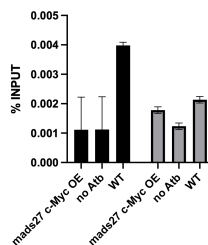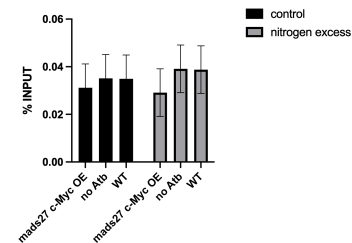

Supplement: Supplementary Figure S1 — HvMiRNA444c targets HvMADS27 transcription factor mRNA. Degradome analysis of 68-day old barley plant grown under control conditions. Perfect complementarity between HvMADS27 mRNA (accession number: HORVU2Hr1G080490.4) and miR444c is shown. The x-axis of the graph represents the length of the HvMADS27 transcript (722 nt), and the y-axis depicts the abundance of identified cleavage and degradation fragments in read numbers. The red line indicates the miRNA444c- mediated cleavage site at position 287. [file Data_Sheet_1.ZIP › Supplementary data/Figure S9.pdf]
