# Supplementary material for: Excess nitrogen responsive HvMADS27 transcription factor controls barley root architecture by regulating abscisic acid level
Source: Front Plant Sci. 2022 Sep 12;13:950796. doi: 10.3389/fpls.2022.950796 (PMC9511987; doi:10.3389/fpls.2022.950796)
Supplement: Supplementary file 3 [file Data_Sheet_2.docx]

**Table S1. Regulatory elements common for *HvMADS27* and *HvMIR444c* gene promotor**

The table depicts *cis*-regulatory elements found in the promoters of both *HvMADS27* and *HvMIR444c* gene promoters, their number, sequence, and function. The left site columns provide motif names, their abundance, sequence, and function, respectively. Red color indicates motifs connected to the N response.

| Motif | Abundance | Sequence | Function | |  |
| --- | --- | --- | --- | --- | --- |
|  | MADS27/MIR444c |  |  |  |  |
| ROOTMOTIFTAPOX1 | 11/6 | ATATT | Root specific expression | |  |
| GATABOX | 16/11 | GATA | GATA motif in CaMV 35S promoter. | | |
|  |  |  | Binding with ASF-2. |  |  |
| GTGANTG10 | 9/16 | GTGA | Motif found in the promoter of the tobacco | | |
|  |  |  | late pollen gene G10 which shows homology | | |
|  |  |  | to pectate lyase and is the | | **putative** |
|  |  |  | homologue of the tomato gene LAT56. | | |
| POLLEN1LELAT52 | 17/7 | AGAAA | One of two co-dependent regulatory | | |
|  |  |  | elements responsible for pollen specific | | |
|  |  |  | activation of tomato LAT52 gene. | |  |
| DOFCOREZM | 31/21 | AAAG | Binding site for Dof proteins | |  |
| CACTFTPPCA1 | 26/24 | YACT | Nitrogen response and component of | | |
|  |  |  | mesophyll expression module 1 found in | | |
|  |  |  | promotor of PHOSPHOENOLPYRUVATE | | |
|  |  |  | CARBOXYLASE (PPCA1) of C4 dicots. | | |
| TBOXATGAPB | 3/3 | ACTTTG | Motif connected to light-activated gene | | |
|  |  |  | expression. |  |  |
| CAATBOX1 | 13/16 | CAAT | Motif found in promoter sequence of LEGA | | |
|  |  |  | gene of pea. |  |  |
| ARR1AT | 11/20 | NGATT | Motif found in promotor of ARR1 gene in | | |
|  |  |  | Arabidopsis. |  |  |
| WRKY71OS | 8/16 | TGAC | Motif found in AMY32B promoter, also a | | |
|  |  |  | binding site of rice WRKY71, a | | |
|  |  |  | transcriptional repressor of the | |  |
|  |  |  | gibberellin signaling pathway. | |  |
| CURECORECR | 14/6 | GTAC | GTAC is the core of a CuRE (copper- | | |
|  |  |  | response element) |  |  |
| EBOXBNNAPA | 20/14 | CANNTG | E-box of NAPA storage-protein gene of | | |
|  |  |  | Brassica napus. |  |  |
| MYCCONSENSUSAT | 20/14 | CANNTG | MYC recognition site found in the | | |
|  |  |  | promoters of the dehydration-responsive | | |
|  |  |  | gene RD22 and many other genes in | | |
|  |  |  | Arabidopsis. |  |  |
| TAAAGSTKST1 | 10/2 | TAAAG | TAAAG motif found in promoter of | | |
|  |  |  | Solanum tuberosum KST1 gene. Target site | | |
|  |  |  | for trans-acting StDof1 protein controlling | | |
|  |  |  | guard cell-specific gene expression. KST1 | | |
|  |  |  | gene encodes a K+ |  |  |
|  |  |  | influx channel of guard cells. | |  |
| GT1CONSENSUS | 13/7 | GRWAAW | Consensus GT-1 binding site in many light- | | |
|  |  |  | regulated genes. |  |  |
| ACGTATERD1 | 4/4 | ACGT | Sequence required for etiolation-induced | | |
|  |  |  | expression of ERD1 (early responsive to | | |
|  |  |  | dehydration) in Arabidopsis. | |  |
| SEF4MOTIFGM7S | 6/2 | CAAACAC | Motif conserved in many storage-protein | | |
|  |  |  | coding genes. |  |  |
| SORLIP1AT | 4/3 | GCCAC | Sequences over-represented in light- | | |
|  |  |  | Inducedpromoters(SORLIPs)in | | |
|  |  |  | Arabidopsis. |  |  |
| NODCON2GM | 3/6 | CTCTT | One of two putative nodulin consensus | | |
|  |  |  | sequences |  |  |
| NODCON1GM | 3/3 | AAAGAT | One of two putative nodulin consensus | | |
|  |  |  | sequences. |  |  |
| OSE1ROOTNODULE | 3/3 | AAAGAT | One of the consensus sequence motifs of | | |
|  |  |  | organ-specific | elements | **(OSE)** |
|  |  |  | characteristic of the promoters activated in | | |
|  |  |  | infected cells of root nodules. | |  |
|  |  |  |  | | |
| DPBFCOREDCDC3 | 4/4 | ACACNNG | A class of bZIP transcription factors, | | |
|  |  |  | DPBF-1 and 2 (Dc3 promoter-binding | | |
|  |  |  | factor-1 and 2) binding core sequence. | | |
|  |  |  | Responsive to ABA. |  |  |
|  |  |  |  | | |
| PRECONSCRHSP70A | 2/5 | SCGAYNRNNNNNNNNNNNNNNNHD | Consensus sequence of PRE (plastid | | |
|  |  |  | response element) in the promoters of | | |
|  |  |  | HSP70A. |  |  |
| BIHD1OS | 4/4 | GTCA | Binding site of OsBIHD1, a rice BELL | | |
|  |  |  | homeodomain transcription factor. | | |
| CCAATBOX1 | 3/4 | CCAAT | Common sequence found in promotor | | |
|  |  |  | regions of genes responsive to heat | |  |

**Table S2. List of primers used in this study**

Table depicts primers used in this study. Primer name, sequence and usage is provided.

| Primer name | Sequence | Use |
| --- | --- | --- |
| AS104 | 5’TCGGTCTCGTCACCTTCTCC3’ | Forward primer. Amplification of MADS27 |
|  |  | mRNA for qPCR study |
| AS105 | 5TTCGCTCGGCCATATCGATC3 | Revers Primer. Amplification of MADS27 |
|  |  | mRNA for qPCR study |
| APO445 | 5’CATGTGAATCTGAGCAATGTGCAAAC3’ | 5’RACE for MIR444c gene |
| APO446 | 5’CTGTCTCTAGCTTGCCGCCTCCACTT3’ | 5’RACE for MIR444c gene |
| APO447 | 5’AGGCCAAAGCAAGCCATCAGATGATAC3’ | 5’RACE for MIR444c gene |
| APO450 | 5’GTTTTCGGGCCATACCGATGATTTTCT3’ | 3’RACE for MIR444c gene |
| APO451 | 5’TGCCTCCCTTTGCCAGAACTATGGAAT3’ | 3’RACE for MIR444c gene |
| APO452 | 5’GAGCCAAAATGCCTCACTCGATTCACT3’ | 3’RACE for MIR444c gene |
| APO453 | 5’GCGCAGGTACATGTTTCCTTAGCTCCA3’ | 3’RACE for MIR444c gene |
| AS3001 | 5’TGAAATGGATATTAGAAATGGG3’ | Forward primer for CHIP-qPCR analysis |
|  |  | for PPC1 carboxylase |
| AS3002 | 5’ATCTTTCATCTCTTCTAGGGTC3’ | Revers primer for CHIP-qPCR analysis for |
|  |  | PPC1 carboxylase |
| AS3003 | 5’AACTGTAGTTTAAAAGTTTTTG3’ | Forward primer for CHIP-qPCR analysis |
|  |  | for first motif in ANR1 promoter |
| AS3004 | 5’GTTATAAAATTGCATAAGTAGCTGC3’ | Revers primer for CHIP-qPCR analysis for |
|  |  | first motif in ANR1 promoter |
| AS3005 | 5’TGATAACAAGATATGATACTATGTG3’ | Forward primer for CHIP-qPCR analysis |
|  |  | for second motif in ANR1 promoter |
| AS3006 | 5’CCTGATTAATTTGATGTTCTTTATGTT3’ | Revers primer for CHIP-qPCR analysis for |
|  |  | second motif in ANR1 promoter |
| AS3007 | 5’CTGTTACAAGGCTTTTGTACGATTC3’ | Forward primer for CHIP-qPCR analysis |
|  |  | for LACASSE |
| AS3008 | 5’GGCAAGTAAGTGGTTAATAATA3’ | Revers primer for CHIP-qPCR analysis for |
|  |  | LACASSE |
| AS3009 | 5’AAGTTGAAGAAACCGTAGCTACAAC3’ | Forward primer for CHIP-qPCR analysis |
|  |  | for UPS2 |
| AS3010 | 5’TTATTAGTAAGCTATGTAC3’ | Revers primer for CHIP-qPCR analysis for |
|  |  | UPS2 |
| AS3011 | 5’ACTGGGTGGATGGTGAAAATGAAC3’ | Forward primer for CHIP-qPCR analysis |
|  |  | for first motif in BG1 promoter |
| AS3012 | 5’TGCGTTTGCTCATTGGAGTTTTATT3’ | Revers primer for CHIP-qPCR analysis for |
|  |  | first motif in BG1 promoter |
| AS3013 | 5’TGCGTCCAACGCTAGCCCGATC3’ | Forward primer for CHIP-qPCR analysis |
|  |  | for second motif in BG1 promoter |
| AS3014 | 5’GCAATTGTGGACGCGTGGACT3’ | Revers primer for CHIP-qPCR analysis for |
|  |  | second motif in BG1 promoter |
| AS916 | 5’TGTACCTTGCGCTGTACCTG3’ | Forward primer used for RT-qPCR analysis |
|  |  | for NRT1.1 |
| AS917 | 5’ACTTCTCGGTGCTGTTGGAC3’ | Revers primer used for RT-qPCR analysis |
|  |  | for NRT1.1 |
| AS942 | 5’GTCCTCGGCCTCTTCCTC3’ | Forward primer used for RT-qPCR analysis |
|  |  | for ABI5 |
| AS943 | 5’CGGTACTGGTGTTGATGGTG3’ | Revers primer used for RT-qPCR analysis |
|  |  | for ABI5 |
| 1CafGNRT1.1F | 5’ATGCCATGCGAGTTACTGGT3’ | Forward primer for CHIP-qPCR analysis |
|  |  | for NRT1.1 promoter |
| 1CARGNRT1.1R | 5’CGGCCACCAAATCAACACTG3’ | Revers primer for CHIP-qPCR analysis for |
|  |  | NRT1.1 promoter |
| APO770 | 5’ATTTGAATTCATGGGGCGGGGCAAGATAGTG3’ | Forward primer for MADS27 CDS |
|  |  | amplification with EcoRI restriction site |
| APO771 | 5’ATTTGGATCCTCATGGATGTAGTTGCAATCCT3’ | Revers primer for MADS27 CDS |
|  |  | amplification with BamHI restriction site |
| APO772 | 5’CACCAACCATGGAGGAGCAGAAGCTGAT3’ | Forward primer used to amplify MADS27 c- |
|  |  | myc from pGBKT7 plasmid |
| HYGR_F | 5’ATTTCGGCTCCAACAATGTC3’ | Forward primer for hygromycing resistance |
|  |  | gene amplification during barley mutants |
|  |  | genotyping |
| HYGR_R | 5’GATGTTGGCGACCTCGTATT3’ | Revers primer for hygromycing resistance |
|  |  | gene amplification during barley mutants |
|  |  | genotyping |
