# Supplementary material for: Excess nitrogen responsive HvMADS27 transcription factor controls barley root architecture by regulating abscisic acid level
Source: Front Plant Sci. 2022 Sep 12;13:950796. doi: 10.3389/fpls.2022.950796 (PMC9511987; doi:10.3389/fpls.2022.950796)
Supplement: Supplementary Figure S1 — HvMiRNA444c targets HvMADS27 transcription factor mRNA. Degradome analysis of 68-day old barley plant grown under control conditions. Perfect complementarity between HvMADS27 mRNA (accession number: HORVU2Hr1G080490.4) and miR444c is shown. The x-axis of the graph represents the length of the HvMADS27 transcript (722 nt), and the y-axis depicts the abundance of identified cleavage and degradation fragments in read numbers. The red line indicates the miRNA444c- mediated cleavage site at position 287. [file Figure S5.pdf › Supplementary data/Figure S5.pdf]

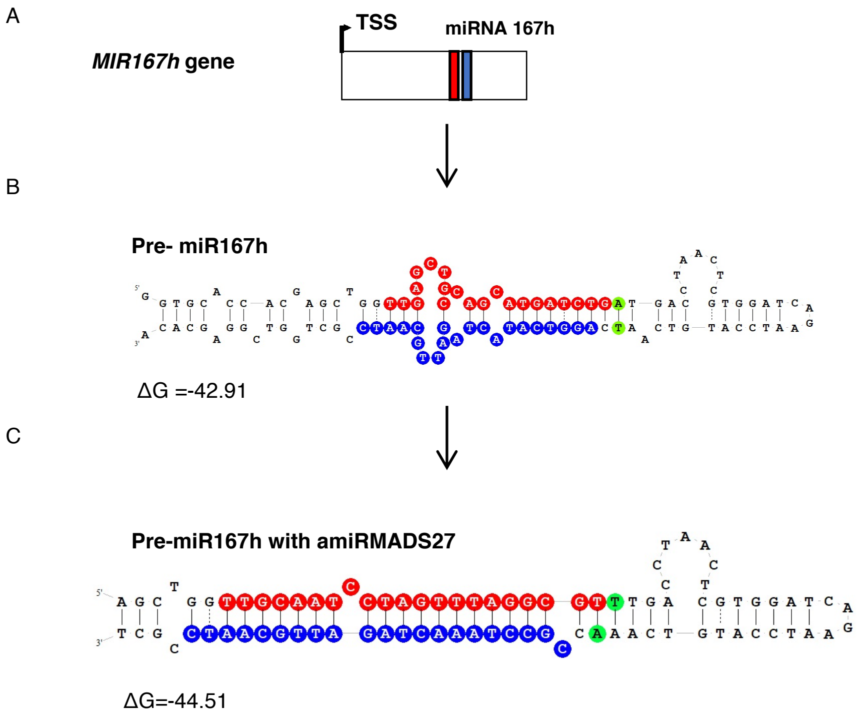
A
TSS
miRNA 167h
MIR167h gene
B
Pre- miR167h
ΔG =-42.91
C
Pre-miR167h with amiRMADS27
ΔG=-44.51
